# Educational disparities in brain health and dementia across Latin America and the United States

**DOI:** 10.1002/alz.14085

**Published:** 2024-08-13

**Authors:** Raul Gonzalez‐Gomez, Agustina Legaz, Sebastián Moguilner, Josephine Cruzat, Hernán Hernández, Sandra Baez, Rafael Cocchi, Carlos Coronel‐Olivero, Vicente Medel, Enzo Tagliazuchi, Joaquín Migeot, Carolina Ochoa‐Rosales, Marcelo Adrián Maito, Pablo Reyes, Hernando Santamaria Garcia, Maria E. Godoy, Shireen Javandel, Adolfo M. García, Diana L. Matallana, José Alberto Avila‐Funes, Andrea Slachevsky, María I. Behrens, Nilton Custodio, Juan F. Cardona, Ignacio L. Brusco, Martín A. Bruno, Ana L. Sosa Ortiz, Stefanie D. Pina‐Escudero, Leonel T. Takada, Elisa de Paula França Resende, Victor Valcour, Katherine L. Possin, Maira Okada de Oliveira, Francisco Lopera, Brian Lawlor, Kun Hu, Bruce Miller, Jennifer S. Yokoyama, Cecilia Gonzalez Campo, Agustin Ibañez

**Affiliations:** ^1^ Latin American Brain Health Institute (BrainLat) Universidad Adolfo Ibañez Santiago Chile; ^2^ Center for Social and Cognitive Neuroscience School of Psychology Universidad Adolfo Ibañez Santiago Chile; ^3^ Cognitive Neuroscience Center Universidad de San Andrés, Ciudad Autónoma de Buenos Aires Buenos Aires Argentina; ^4^ Consejo Nacional de Investigaciones Científicas y Técnicas (CONICET), Ciudad Autónoma de Buenos Aires Buenos Aires Argentina; ^5^ Department of Neurology Harvard Medical School Boston Massachusetts USA; ^6^ Global Brain Health Institute (GBHI) Trinity College Dublin Dublin Ireland; ^7^ Universidad de los Andes Bogotá D.C. Colombia; ^8^ Global Brain Health Institute University of California San Francisco California USA; ^9^ Centro Interdisciplinario de Neurociencia de Valparaíso (CINV) Valparaíso Chile; ^10^ Departamento de Física Universidad de Buenos Aires, Ciudad Autónoma de Buenos Aires Buenos Aires Argentina; ^11^ Instituto de Física de Buenos Aires (FIBA –CONICET), Ciudad Autónoma de Buenos Aires Buenos Aires Argentina; ^12^ Instituto de Envejecimiento, Facultad de Medicina, Pontificia Universidad Javeriana Bogotá D.C. Colombia; ^13^ Center for Memory and Cognition, Hospital Universitario San Ignacio Bogotá, San Ignacio Bogotá D.C. Colombia; ^14^ Memory and Aging Center Department of Neurology University of California San Francisco California USA; ^15^ Departamento de Lingüística y Literatura Facultad de Humanidades Universidad de Santiago de Chile Santiago Chile; ^16^ Dirección de Enseñanza Instituto Nacional de Ciencias Médicas y Nutrición, Salvador Zubirán Ciudad de México D.C. México; ^17^ Geroscience Center for Brain Health and Metabolism (GERO) Santiago Chile; ^18^ Memory and Neuropsychiatric Center (CMYN) Neurology Department Hospital del Salvador & Faculty of Medicine University of Chile Santiago Chile; ^19^ Neuropsychology and Clinical Neuroscience Laboratory (LANNEC) Physiopathology Program – Institute of Biomedical Sciences (ICBM) Neuroscience and East Neuroscience Departments Faculty of Medicine University of Chile Santiago Chile; ^20^ Servicio de Neurología, Departamento de Medicina Clínica Alemana‐Universidad del Desarrollo Santiago Chile; ^21^ Faculty of Medicine University of Chile Santiago Chile; ^22^ Centro de Investigación Clínica Avanzada (CICA), Universidad de Chile Santiago Chile; ^23^ Unit Cognitive Impairment and Dementia Prevention Peruvian Institute of Neurosciences Lima Peru; ^24^ Facultad de Psicología Universidad del Valle Cali Colombia; ^25^ Departamento de Psiquiatría y Salud Mental Facultad de Medicina Universidad de Buenos Aires, Ciudad Autónoma de Buenos Aires Buenos Aires Argentina; ^26^ Instituto de Ciencias Biomédicas Universidad Católica de Cuyo San Juan Argentina; ^27^ Instituto Nacional de Neurología y Neurocirugía Ciudad de México D.C. México; ^28^ Universidade de São Paulo São Paulo Brazil; ^29^ Universidade Federal de Minas Gerais Belo Horizonte Minas Gerais Brazil; ^30^ Cognitive Neurology and Behavioral Unit (GNCC) University of São Paulo São Paulo Brazil; ^31^ Neurosicence Research Group (GNA) Universidad de Antioquia Medellín Antioquia Colombia; ^32^ Department of Anesthesia, Critical Care and Pain Medicine Massachusetts General Hospital, Harvard Medical School Boston Massachusetts USA

**Keywords:** dementia, educational disparities, healthy aging, Latin America, magnetic resonance imaging, United States

## Abstract

**BACKGROUND:**

Education influences brain health and dementia. However, its impact across regions, specifically Latin America (LA) and the United States (US), is unknown.

**METHODS:**

A total of 1412 participants comprising controls, patients with Alzheimer's disease (AD), and frontotemporal lobar degeneration (FTLD) from LA and the US were included. We studied the association of education with brain volume and functional connectivity while controlling for imaging quality and variability, age, sex, total intracranial volume (TIV), and recording type.

**RESULTS:**

Education influenced brain measures, explaining 24%–98% of the geographical differences. The educational disparities between LA and the US were associated with gray matter volume and connectivity variations, especially in LA and AD patients. Education emerged as a critical factor in classifying aging and dementia across regions.

**DISCUSSION:**

The results underscore the impact of education on brain structure and function in LA, highlighting the importance of incorporating educational factors into diagnosing, care, and prevention, and emphasizing the need for global diversity in research.

**Highlights:**

- Lower education was linked to reduced brain volume and connectivity in healthy controls (HCs), Alzheimer's disease (AD), and frontotemporal lobar degeneration (FTLD).
- Latin American cohorts have lower educational levels compared to the those in the United States.
- Educational disparities majorly drive brain health differences between regions.
- Educational differences were significant in both conditions, but more in AD than FTLD.
- Education stands as a critical factor in classifying aging and dementia across regions.

## 1 BACKGROUND

Formal educational attainment is an important indicator of socioeconomic disparities,^1^, ^2^, ^3^ significantly influencing brain health^3^, ^4^, ^5^, ^6^ and dementia.^7^, ^8^, ^9^, ^10^ Education also fosters brain structure and function, shaping the gray matter volume^11^, ^12^ and connectivity^13^, ^14^ of the frontal, temporal, and occipitoparietal areas. Individuals who have achieved higher education tend to have a lower incidence and delayed onset of dementia.^9^, ^15^ Compensatory educational mechanisms may mitigate tau and amyloid beta aggregation in those key brain areas or primary targets in neurodegeneration.^16^, ^17^ Thus, higher education is associated with reduced risks, prevalence, and delayed cognitive decline in Alzheimer's disease (AD) and frontotemporal lobar degeneration (FTLD).^9^, ^18^

Latin America (LA) is characterized by larger socioeconomic disparities, leading to a higher prevalence of dementia compared to the United States (US).^7^, ^19^ Latinos face a larger prevalence of dementia, a problem set to escalate in the upcoming decades,^20^ influenced by limited educational opportunities.^8^, ^19^ Nonetheless, previous reports on dementia, including education measures, have multiple gaps. First, the effects of education on brain health and dementia have been predominantly examined only as a protective factor in epidemiological studies or as a confounder in group comparisons.^7^, ^9^ Second, the impact of education on brain structure and function in the context of dementia has rarely been evaluated.^21^, ^22^ Third, there are no comparative data on the effect of education on brain structure and function across diverse geographical populations with varying levels of socioeconomic disparities, including LA.^23^ Thus, it remains unknown whether education affects brain structure and function differently in aging and dementia in LA compared to the US. Filling these knowledge gaps is important to understand the differences in dementia presentation between these regions^7^, ^18^, ^19^, ^23^ and the potential variable impacts on different dementia types (AD and FTLD).

In this study, we aimed to elucidate the impact of educational disparities on the brain structure and function of healthy controls (HCs) and individuals with dementia (AD and FTLD) across LA and the US in two geographical samples exhibiting comparable levels of cognitive impairment. We assessed gray matter volume and network measurements through voxel‐based morphometry and resting‐state functional connectivity, respectively. We addressed potential confounders related to imaging quality and inter‐scan variability while controlling for age, sex, total intracranial volume, and type of resting‐state recording. First, we assessed geographical variations in the brain correlates of education. Subsequently we explored the geographical differences explained by education on whole‐brain analysis across conditions. Finally, employing machine learning algorithms, we disentangled the contributions of demography, education, cognition, and measures of brain structure and function to a multiclass classification (i.e., each group against all others) of conditions (HCs, AD, FTLD) and geographical regions (LA and US). Using a large sample size (*n* = 1412), the multiclass algorithms were trained with 80% of the data and tested with the remainder, following a 5‐fold, cross‐validation procedure for robust model assessment. Considering the pronounced economic disparities in LA, we hypothesized a substantial influence of education on brain structure and function with a specific gradient depending on geographical distribution (LA > US) and condition (HCs < AD = FTLD). We also hypothesized that education would emerge as a top predictor in the multiclass group characterization, encompassing demography, cognition, gray matter volume, and functional connectivity. The Alzheimer's Disease Neuroimaging Initiative (ADNI) has been the cornerstone of this project, augmented by data sets from additional initiatives. We anticipate that our results will have significance for understanding phenotypical heterogeneity in healthy aging and dementia^24^, ^25^, ^26^ and its social determinants,^2^, ^27^ providing valuable insights for developing tailored models and personalized care.^19^

## 2 MATERIAL AND METHODS

### 2.1 Participants

This cross‐sectional observational study comprised 1412 participants (mean_age_ = 66.8, SD_age_ = 9.4; 55.8% women) from LA and the US, with 625 HCs with preserved cognition (Mini‐Mental State Exam, MMSE > 24 points) and no antecedents of psychiatric and neurological conditions. These antecedents were verified through a structured clinical interview, confirming that none of the HCs had any relatives with a history of neurodegenerative diseases. A total of 385 participants met the criteria for probable AD.^28^ A group of 402 subjects with FTLD^29^ met diagnostic criteria for different variants, including behavioral variant frontotemporal dementia,^30^ primary progressive aphasia,^31^ and motor syndromes.^32^, ^33^ LA participants were recruited from the Multi‐Partner Consortium to Expand Dementia Research in Latin America (ReDLat, with participants from Mexico, Colombia, Peru, Chile, and Argentina).^34^ US participants were non‐Latino individuals from ReDLat, ADNI,^35^ and the neuroimaging in frontotemporal dementia (NIFD)^36^ repositories. To ensure data reliability, we excluded subjects who reported a history of alcohol/drug abuse or psychiatric or other neurological illnesses. Supporting the clinical criteria, each dementia diagnosis presented its expected atrophy pattern (Supplementary Material 1). ReDLat gathers data from different centers across countries, utilizing a standardized data framework and harmonized diagnostic protocols (e.g.,^19^, ^37^, ^38^, ^39^, ^40^). Exclusion criteria include participants with syndromes other than AD, FTLD, or impairments that prevent task completion. Diagnosis is determined by consensus among expert groups^41^, ^42^ at each site, taking into account cognitive and neurological examinations, clinical interviews, and magnetic resonance imaging (MRI).^43^ A standardized battery captures clinicians’ evaluations.^44^ Both clinical and cognitive assessments across ReDLat sites are harmonized, normalized, and validated.^40^ All clinicians undergo training and certification by a specialized team^34^ and adhere to a quality control protocol. Demographic variables on each condition and region for both T1‐weight and resting‐state functional MRI (rs‐fMRI) samples are shown in Table 1 (more details in Supplementary Material 2). Education was measured as the number of schooling years completed satisfactorily in any country. All participants provided written informed consent in agreement with the Declaration of Helsinki. The institutional review boards from each recruitment site and the Executive Committee of ReDLat approved the study procedures.

**RESEARCH IN CONTEXT**

1. **Systematic review**: The impact of education disparities on brain structure and function, particularly in dementia, is not yet understood. Latin America (LA) experiences more pronounced socioeconomic disparities and limited educational opportunities compared to the United States (US), potentially contributing to the higher prevalence of dementia in LA than in the US.
2. **Interpretation**: Results showed a robust effect of education on aging and dementia, especially in LA. This calls for incorporating education into strategies for diagnosing, managing, and preventing dementia. Specifically, improved public policies in LA must increase educational levels to reduce the impact of dementia.
3. **Future directions**: Our research underscores the need to increase the diversity of registries in dementia worldwide, analyzing the geographical differences between populations and highlighting the impact of education on these discrepancies. The Alzheimer's Disease Neuroimaging Initiative (ADNI) could explore similar effects across other regions, emphasizing the requirement for globally adaptable study designs.

**TABLE 1 alz14085-tbl-0001:** Demography and cognition of subjects in the T1 and rs‐fMRI datasets.

	HC (LA/US)	AD (LA/US)	FTLD (LA/US)
	T1		
Sample size	318/307	193/192	208/194
Age (mean years)	64.6/69.5^a^	72.2/67.2^a^	65.8/63.7^a^
Sex (no. female)	205/175^a^	129/106	102/78
Education (mean years of education attainment)	15.1/17.2^a^	10.5/16.3^a^	12.9/16.3^a^
Cognition (mean MMSE)	28.6/28.1	18.7/17.8	22.3/22.5
	rs‐fMRI		
Sample size	240/332	168/152	177/194
Age (mean years)	67.5/62.8^a^	72.4/67.9^a^	66.2/63.7^a^
Sex (number of females)	159/184^a^	114/75^a^	87/77
Education (mean years of education attainment)	15.4/16.9^a^	10.9/16.3^a^	12.7/16.2^a^
Cognition (mean MMSE)	28.7/27.9	20.4/19.9	22.3/22.6

*Note*: Kruskal–Wallis test was used to compare continuous variables, as the data were non‐normally distributed (Shapiro–Wilk test *p* < 0.001). The *Χ* ^2^ test for equality of proportions with continuity correction was used to compare sex variables. All *p*‐values were adjusted using Bonferroni correction and set to *p* < 0.05.

Abbreviations: AD, Alzheimer's disease; FTLD, frontotemporal lobar degeneration; HCs, healthy controls; LA, Latin American; MMSE, Mini‐Mental State Exam; US, United States.

^a^ Significant differences in the comparison between geographical regions (LA vs. US).

### 2.2 MRI acquisition

Images were obtained from 16 different scanner models (Table S3), following ADNI protocols. Whole‐brain structural T1‐weighted and resting‐state sequences were registered in the whole sample (*n* = 1412) and from a subset of participants (*n* = 1263), respectively.[Table alz14085-tbl-0001] To ensure quality, all images were carefully selected and confirmed by visual inspection and quantitative metrics calculated using MRIQC^44^ (see Supplementary Material 4 for further details). We included two resting‐state recordings, closed and open eyes, to increase the sample size for rs‐fMRI data. The potential impact of the scanner models on our analyses was assessed through machine learning algorithms. The scanner model did not affect the multiclass classification of conditions (Supplementary Material 3). In addition, we controlled variability using another approach. Because site harmonization can affect results due to scanner variability across geographic regions, we implemented an intra‐subject harmonization for each modality. In previous work, we have combined data from ReDLat with ADNI databases, showing adequate comparability.^45^

### 2.3 MRI preprocessing

T1‐weighted images were pre‐processed and analyzed using the voxel‐based morphometry method with the Computational Anatomy (CAT12) toolbox (https://neuro‐jena.github.io/cat/) in Matlab R2022a. The pre‐processing pipeline included bias‐field correction, noise reduction, skull stripping, segmentation, and normalization to the Montreal Neurological Institute (MNI) space at a 0.5 mm isotropic resolution, following the default parameters of the toolbox. CAT12 applies an intra‐subject harmonization approach based on normalizing the data to the mean global intensity for each subject. Subsequently, gray matter segmentations underwent smoothing with a Gaussian kernel of 6 × 6 × 6 mm. The homogeneity and orthogonality of the resulting samples were thoroughly verified.

Preprocessing of rs‐fMRI was conducted using the fmriprep (version 22.0.2) standard pipeline to ensure replicability.^46^ Further steps and analyses were performed using the CONN22.a toolbox.^47^ CONN pre‐processing involved smoothing with a Gaussian kernel of 6 × 6 × 6 mm, de‐noising through linear regression to account for confounding effects of white matter, cerebrospinal fluid, realignment, and scrubbing. A band‐pass filter within the frequency range of 0.008–0.09 Hz was also applied. The global correlation measure was adopted to characterize brain connectivity, quantifying the average correlation coefficient between each voxel and the rest of the brain.^47^ To enhance comparability in functional measures, we implemented an inter‐subject harmonization, which normalized voxel‐level values for each subject into a Gaussian distribution with a mean of 0 and a SD of 1.^47^

We employed region‐of‐interest (ROI) analysis based on the automated anatomic labeling (AAL) atlas^48^ to reduce the dimensionality of MRI data for machine learning algorithms. For structural data, we calculated the volume divided by the total intracranial volume (TIV) for each ROI within the AAL. The mean Pearson correlation was computed for each ROI with the rest of the ROIs in functional data. This method was used previously at the voxel level in functional connectivity analysis.^47^

### 2.4 Statistical analysis

To assess the potential effects of heterogeneity, our study employed three analyses: regression, geographic comparisons by groups, and multiclass classifications. All these were corrected for age and sex. The sample size in all analyses allowed us to achieve a power analysis of 0.99, calculated with G power software to detect a large effect size (δ > 0.8) with a *p* < 0.001.

#### 2.4.1 Impact of education on gray matter volume across conditions and geographical regions

##### Structural correlates of education

To investigate the impact of education, we began by examining its neural correlates and how they change across geographical regions. We focused on the associations between gray matter volume and education. For this, we conducted a voxel‐based morphometry regression analysis using the entire sample while controlling for age, sex, and TIV. All analyses were corrected via the threshold‐free cluster enhancement (TFCE) method^49^ for multiple comparisons, utilizing the TFCE toolbox (http://www.neuro.uni‐jena.de/tfce). This method integrates voxel and cluster thresholds, thereby eliminating the need for arbitrary thresholds to form clusters. It also offers heightened sensitivity to both focal and peripheral effects compared to traditional correction methods, striking an optimal balance between familywise error (FWE) rates and replicability.^49^ The statistical significance was determined through 5000 permutations and set at *p*  <  0.05 (FWE‐corrected). Then, statistical parameters changed from *t* to TFCE distributions.^49^ After identifying the structural correlates of education, we examined their differences between the LA and the US participants. We employed the significant areas from the initial analysis as a mask to calculate the individual volume within these areas; these volume values were then normalized using a min–max scale and adjusted for TIV to avoid potential confounding due to variations in head size. Subsequently, we compared LA and the US subjects across conditions through a Kruskal–Wallis test; in addition, we used 5000 bootstrap resamples to calculate the mean differences. To assess the impact of cultural differences, we compared each LA country with the US (Supplementary Material 5).

##### Geographical differences in structural whole‐brain analysis

We assessed the amount of variance explained by education in the geographical differences. Specifically, within each group (HCs, AD, and FTLD), we first compared whole‐brain gray matter volume between geographical regions (LA vs US) while controlling for age, sex, and TIV. Then, we introduced education as an additional controlling variable in a subsequent comparison. To evaluate the association in both comparisons, we compared the whole‐brain β values across conditions using a Kruskal–Wallis test.^50^ These coefficients indicated the extent to which gray matter volume is expected to change after each subsequent year of education while holding the other variables constant. In line with standard practice to increase reliability,^50^ these values were calculated for each voxel that presented a significant difference in the geographical comparisons. To measure the variance explained by education, we calculated the differences in the statistical values at the voxel level between both comparisons across conditions. Use of the TFCE method to account for multiple comparisons imposed the use of the TFCE values as the result of these comparisons. We calculated the variance on the whole‐brain TFCE values of the uncontrolled comparisons and the subtraction. The geographical differences explained by education were measured by calculating the percentage represented by the variance of the subtractions in the variance of the uncontrolled comparisons. We also did the same operation to extract the variance explained by education within the frontal, temporal, parietal, and occipital lobes. Furthermore, we analyzed the anatomic structures implicated in the map of the resulting subtractions.

#### 2.4.2 Impact of education on functional connectivity across conditions and geographical regions

The functional connectivity analysis followed the same steps as the gray matter analysis. In these analyses, global correlation^47^ served as the dependent variable. Age, sex, and the type of resting‐state recording e controlled as covariates. A global correlation index represented the average correlation coefficient between each voxel and all other voxels in the brain,^47^ and the type of resting‐state recording was codified as a dummy variable (open or closed eyes).

#### 2.4.3 Multiclass classification

We assessed the contribution of demography, education, gray matter volume, and functional connectivity to a multiclass classification^51^ between groups (across conditions and geographical regions). An XGBoost multiclass classification targeted the interaction between conditions and geographical regions (six groups). This classification was accomplished using a one‐vs‐rest strategy,^52^ in which each group (each geographical condition) was individually classified against the remaining groups. To ensure a robust model assessment, a 5‐fold, cross‐validation procedure was implemented using 80% of the available data for training.^52^ The multi‐logloss metric was used during model training to assess classification error.^52^ The model parameter optimization involved a Bayesian hyperparameter tuning, a probabilistic model‐based search method.^52^ Bootstrap resampling techniques were employed to estimate the variance in the multiclass receiver‐operating characteristic (ROC) curves. The performance was evaluated by calculating the area under the curve (AUC) score, accuracy, sensitivity, specificity, precision, recall, and F1 for each target group relative to all others. Finally, an in‐depth feature importance analysis was conducted through bootstrapped feature lists. This method involved creating numerous subsets of the original data set through bootstrapping with random sampling and replacement. A separate instance of the classification model was trained on each of these subsets, allowing the evaluation of their feature importance within several data sets. By repeatedly introducing models on these bootstrapped samples, it was possible to calculate the importance of each feature. The important scores derived from this process were averaged to determine a stable estimate. This step aided in classifying the order of relevance of the implemented features.

We adopted two models to classify groups. The first model included gray matter volume data for each ROI defined in the AAL atlas.^48^ The second model used functional connectivity data from the same atlas. We calculated the average per ROI of the functional connectivity matrix^47^ to have two comparable models regarding the number of features. In both cases, we included cognition (as measured by MMSE scores), demography (age and sex), education, and, in the case of functional connectivity, the type of resting‐state recording (dummy).

## 3 RESULTS

### 3.1 Demography

Formal educational attainment was significantly lower in LA than in the US across all conditions (Table 1). Geographical comparisons (LA vs US) revealed differences in mean age in all conditions, and the majority exhibited imbalances in sex distribution. Conversely, no significant differences were observed in cognition between the LA and the US group's HCs, AD, and FTLD pairs. However, as expected, cognition significantly differed when comparing AD and FTLD against the HCs in both geographic regions (Supplementary Material 6).

### 3.2 Education impacts gray matter volume across conditions and geographical regions

#### 3.2.1 Structural correlates of education

**FIGURE 1:**
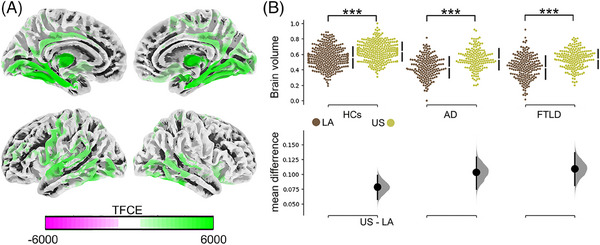
Structural correlates of education differ between geographical regions. (A) Brain maps showing the association of brain volume with educational attainment, controlled by age and sex. Multiple comparisons were corrected with the threshold‐free cluster method with a *p*_FWE_ < 0.05. (B) Top panel: Scatter plots showing geographical comparisons across conditions for brain volume within areas positively associated with educational attainment. Comparisons were computed using the Kruskal–Wallis test. Bottom panel: The effect size of the geographical comparison across conditions. We utilized 5000 bootstrap resamples to calculate the mean differences. TFCE method to account for familywise error was used to correct multiple comparisons. AD, Alzheimer's disease; FTLD, frontotemporal dementia with lobar degeneration; HCs, healthy control; LA, Latin America; TFCE, threshold‐free cluster.

Associations between higher education and larger gray matter volume were significant with temporal areas, including the hippocampus and inferior and superior temporal gyri. Similar associations were observed in the posterior cingulum and the orbitofrontal cortices (Figure 1A; more details in Supplementary Material 7). Comparative analysis within these significant areas revealed consistent volume reduction in individuals from LA compared to those from the US: Stats_HC_: *Χ* ^2 ^= 23, *p* < 0.001; Stats_AD_: *Χ* ^2 ^= 17, *p* < 0.001; Stats_FTLD_: *Χ* ^2 ^= 21.5, *p* < 0.001 (Figure 1B, top panel), with higher mean differences in AD and FTLD than in HCs (Figure 1B, bottom panel). All analyses were controlled for age, sex, and TIV. These results remain consistent even when accounting for intercultural effects by comparing the US against each LA country (Supplementary Material 5).

#### 3.2.2 Geographical differences in structural whole‐brain analysis explained by education

The correction through education in the geographical comparisons of whole‐brain gray matter volume diminished the geographical differences (comparisons adjusted for age, sex, and TIV). A more robust association was evident in the controlled than uncontrolled education comparisons, as indicated by the higher *β* values in the controlled comparison. This pattern was observed across all conditions (Stats_HC_: *Χ* ^2 ^= 84, *p* < 0.001; Stats_AD_: *Χ* ^2 ^= 411, *p* < 0.001; Stats_FTLD_: *Χ* ^2 ^= 84.2, *p* < 0.001). The results from these comparisons showed smaller sizes in the significant clusters of the corrected comparisons (Supplementary Material 7).

**FIGURE 2:**
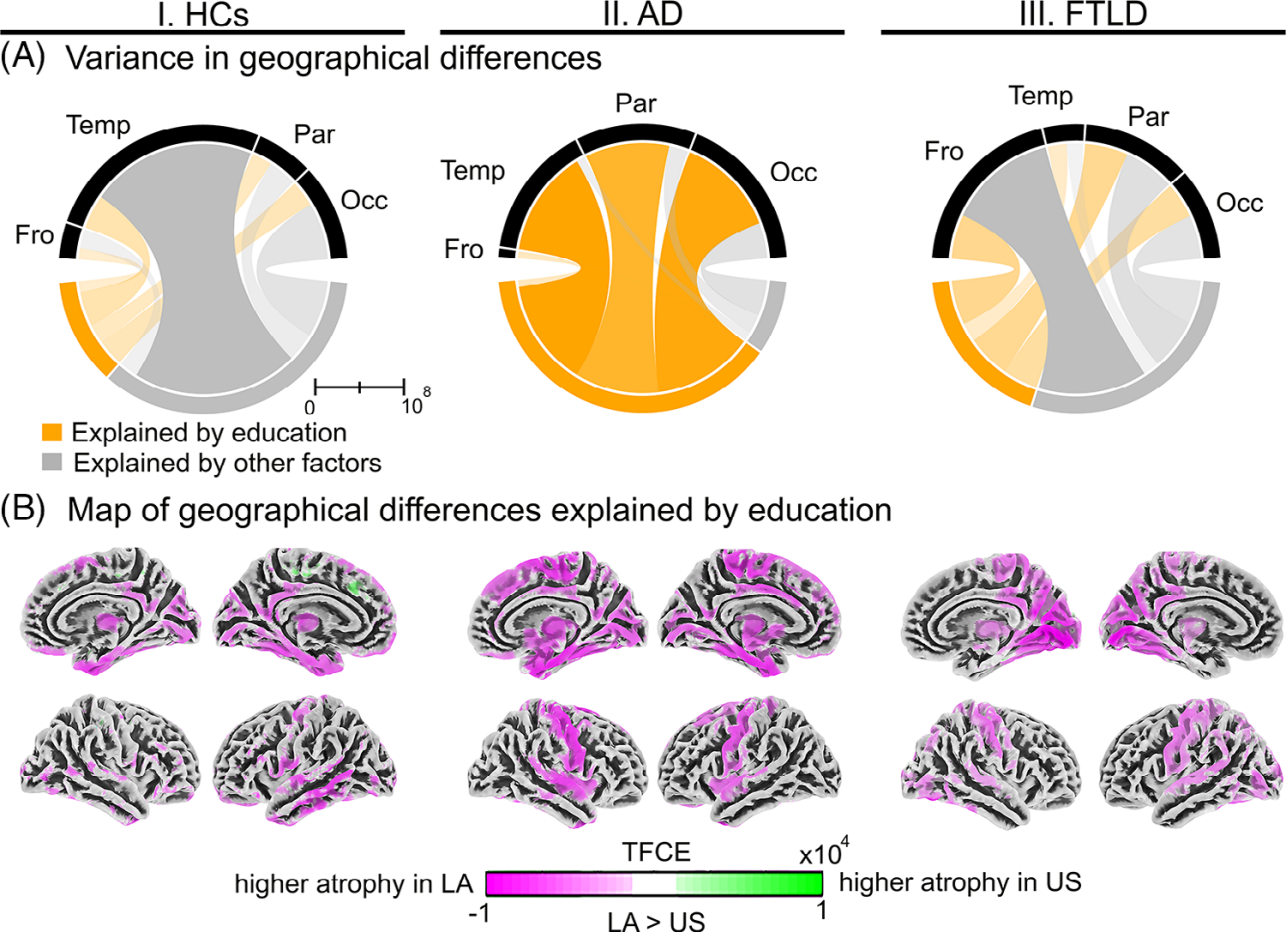
Geographical differences (LA v. the US) explained by education in gray matter volume across conditions. Sections I, II, and III present information regarding HCs, AD, and FTLD, respectively. (A) Variance of the TFCE values within the cerebral lobes explained by education or other factors. All analyses were adjusted for age, sex, and total intracranial volume. (B) Map of the geographical differences explained by education. The image represented the subtraction of the TFCE values at the voxel level from two comparisons between (LA vs the US), controlled and uncontrolled through education. In these comparisons, the TFCE method accounted for familywise errors to correct multiple comparisons. AD, Alzheimer's disease; Fro, frontal; FTLD, frontotemporal lobar degeneration; HCs, healthy controls; LA, Latin America; Occ, occipital; Par, parietal; Temp, temporal; TFCE, threshold‐free cluster enhancement; US, United States.

To examine the geographical differences explained by education, we subtracted the TFCE values at the voxel level between the two comparisons across conditions. The percentage of the variances in geographical differences explained by education were 24.6%, 65.0%, and 42.4% in HCs, AD, and FTLD, respectively. Figure 2A showed these variances within the frontal, temporal, parietal, and occipital lobes across conditions. The maps resulting from the previous subtractions showed lower gray matter volume in the LA sample across all conditions (Figure 2B). In HCs, geographical differences explained by education were identified in the temporal pole, middle and inferior temporal gyri, precuneus, posterior cingulate, primary motor, and the occipital cortices (Figure 2B, panel I; details in Supplementary Material 7). In AD, differences depending on education appeared in the superior and inferior temporal gyri, the motor cortex, and occipital areas (Figure 2B, panel II; details in Supplementary Material 7). In FTLD, these differences included posterior temporal regions, occipital cortex, precuneus, posterior cingulum, and the primary motor cortex (Figure 2B, panel III**;** details in Supplementary Material 7).

### 3.3 Education impacts functional connectivity across conditions and geographical regions

#### 3.3.1 Functional correlates of education

**FIGURE 3:**
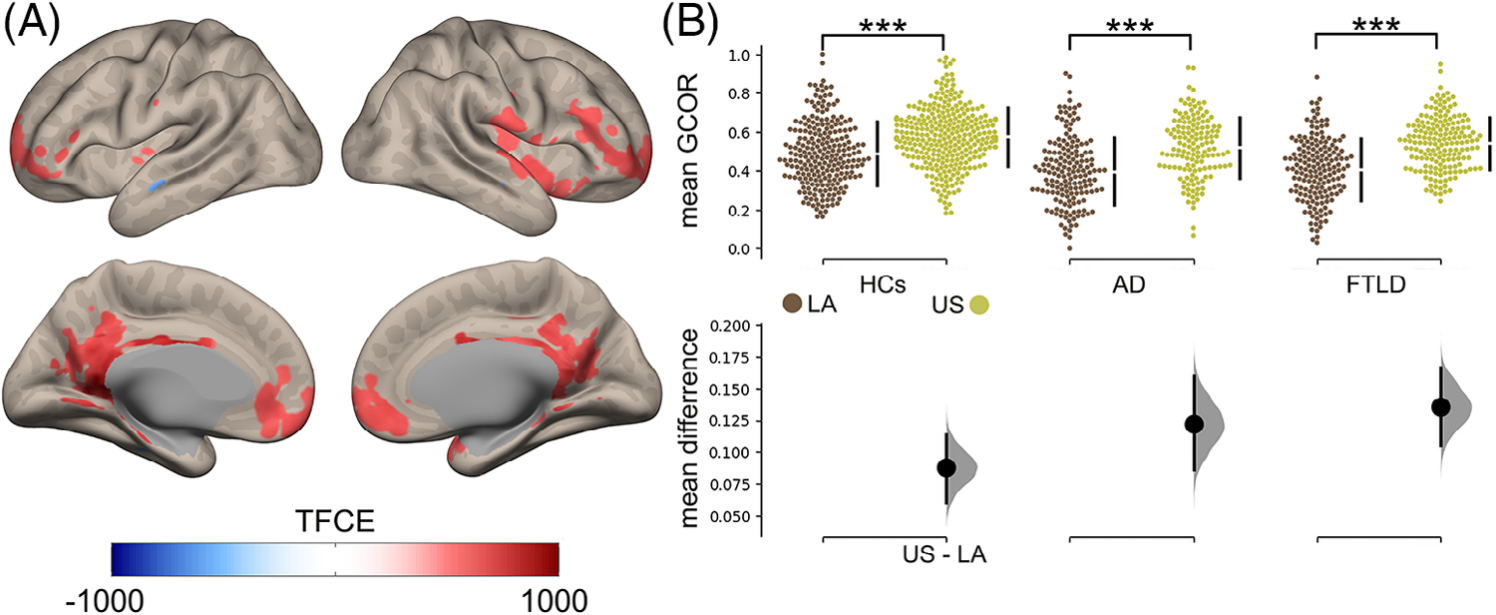
Functional correlates of education differ between geographical regions. (A) Brain maps showing the association between educational attainment and functional connectivity, controlled by age and sex. Multiple comparisons were corrected with the TFCE method with a *p_FWE_* < 0.05. (B) Top panel: Scatter plots showing geographical comparisons across conditions for mean functional connectivity within areas associated with educational attainment. Comparisons were computed using the Kruskal–Wallis test. Bottom panel: The effect size of the geographical comparison across conditions. We utilized 5000 bootstrap resamples to calculate the mean differences. TFCE method to account for familywise error was used to correct multiple comparisons. AD, Alzheimer's disease; FTLD, frontotemporal lobar degeneration; GCOR, global correlation; HCs, healthy controls; LA, Latin America; TFCE, threshold‐free cluster.

Individuals with higher education exhibited increased functional connectivity in key brain areas, including the orbitofrontal cortex, precuneus, posterior cingulum, hippocampus, right insula, and the right superior temporal gyrus (Figure 3A **;** details in Supplementary Material 7). Within these areas, the mean functional connectivity was higher in US individuals than in those from LA. This pattern remained consistent across conditions: Stats_HCs_: *Χ* ^2 ^= 22, *p* < 0.001; Stats_AD_: *Χ* ^2 ^= 21, *p* < 0.001; Stats_FTLD_: *Χ* ^2 ^= 28, *p* < 0.001 (Figure 3B, top panel). However, the effect sizes, as quantified by the mean differences, were higher in patients with AD and FTLD compared to HCs (Figure 3B, bottom panel). All analyses were controlled for age, sex, and type of resting‐state recording. These findings stay stable after analyzing the intercultural effect and comparing the US and each LA country (Supplementary Material 5).

#### 3.3.2 Geographical differences in functional whole‐brain analysis explained by education

**FIGURE 4:**
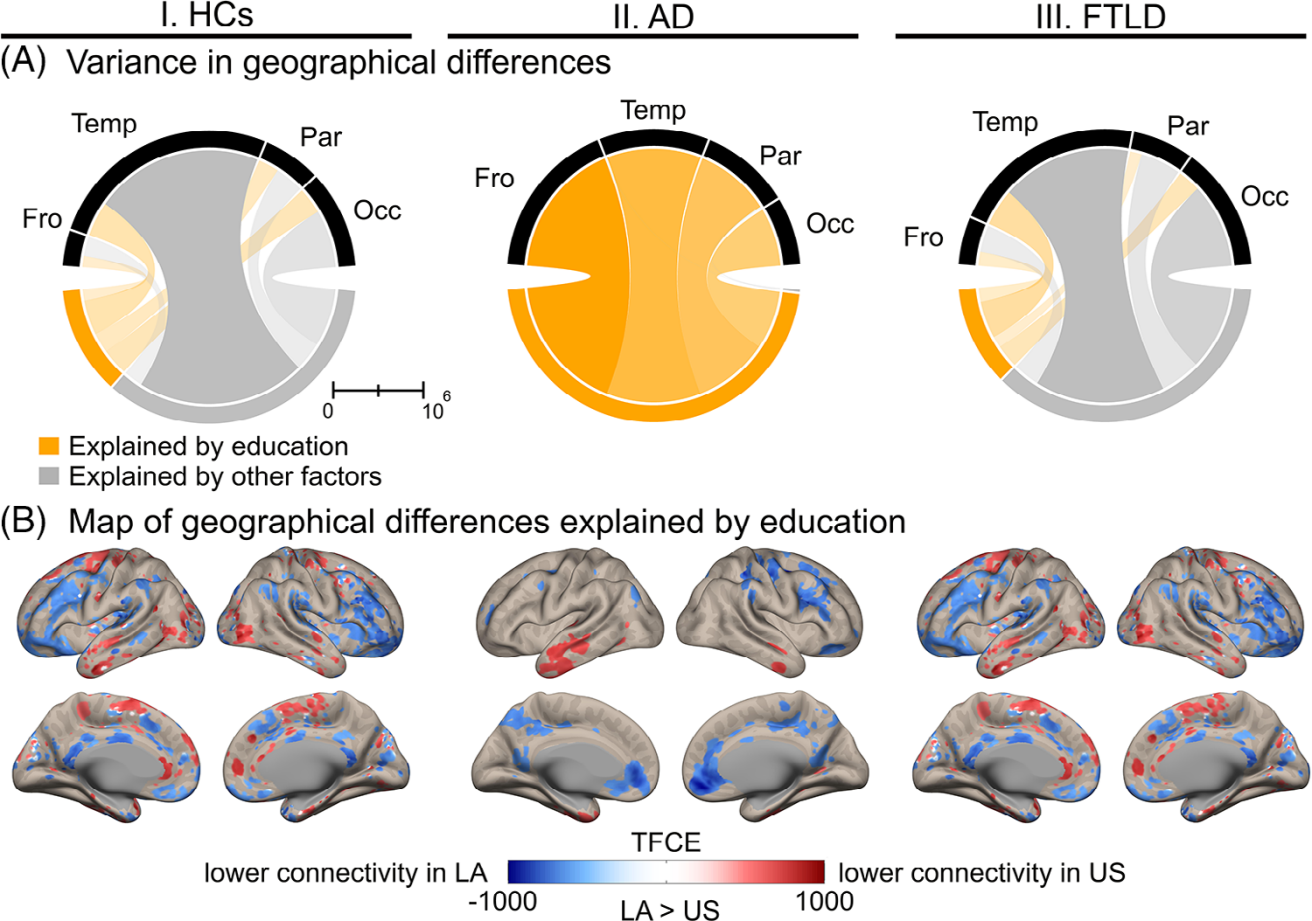
Geographical differences (LA vs the US) explained by education in functional connectivity across conditions. Sections I, II, and III presented information regarding HCs, AD, and FTLD, respectively. (A) Variance of the TFCE values within the cerebral lobes explained by education or other factors. All analyses were adjusted for age, sex, and type of resting‐state recording. (B) Map of the geographical differences explained by education. The image represented the subtraction of the TFCE values at the voxel level from two comparisons between (LA vs the US), controlled and uncontrolled through education. In these comparisons, the TFCE method accounted for familywise errors to correct multiple comparisons. AD, Alzheimer's disease; Fro, frontal; FTLD, frontotemporal lobar degeneration; HCs, healthy controls; LA, Latin America; Occ, occipital; Par, parietal; Temp, temporal; TFCE, threshold‐free cluster enhancement; US, United States.

Correcting for education in geographical comparisons of whole‐brain functional connectivity reduced the differences between LA and the US (all comparisons were adjusted for age, sex, and type of resting‐state recording). Consistently, the *β* values were higher for comparisons controlled than uncontrolled through education, showing a better association in the first‐mentioned comparison. This pattern was observed consistently across all conditions (Stats_HC_: *Χ* ^2 ^= 56, *p* < 0.001; Stats_AD_: *Χ* ^2 ^= 567, *p* < 0.001; Stats_FTLD_: *Χ* ^2 ^= 76.8, *p* < 0.001). Results from both comparisons showed smaller sizes in the significant clusters of the corrected comparisons (Supplementary Material 7). The total variance explained by education were 30.0%, 98.7%, and 27.0% in HCs, AD, and FTLD, respectively. Figure 4A showed this variance within the frontal, temporal, parietal, and occipital lobes across conditions. The TFCE values resulted from the subtraction between uncontrolled and controlled through education comparisons included both negative and positive values (Figure 4B). In HCs, the geographical differences explained by education had lower connectivity in the orbitofrontal and the insular area and higher connectivity in the temporal and occipital regions in the LA sample (Figure 4B, panel I**;** details in Supplementary Material 7). In AD, these differences for LA subjects comprised lower connectivity in the orbitofrontal and precuneus areas but higher connectivity in the temporal pole (Figure 4A, panel II**;** details in Supplementary Material 7). Similarly, FTLD patients from LA exhibited geographical differences explained by education with lower connectivity in the insula, precuneus, and posterior cingulum but higher in the temporal pole, dorsolateral, and occipital areas (Figure 4A, panel III; details in Supplementary Material 6).

### 3.4 Education is a critical contributor to multiclass classification

**FIGURE 5:**
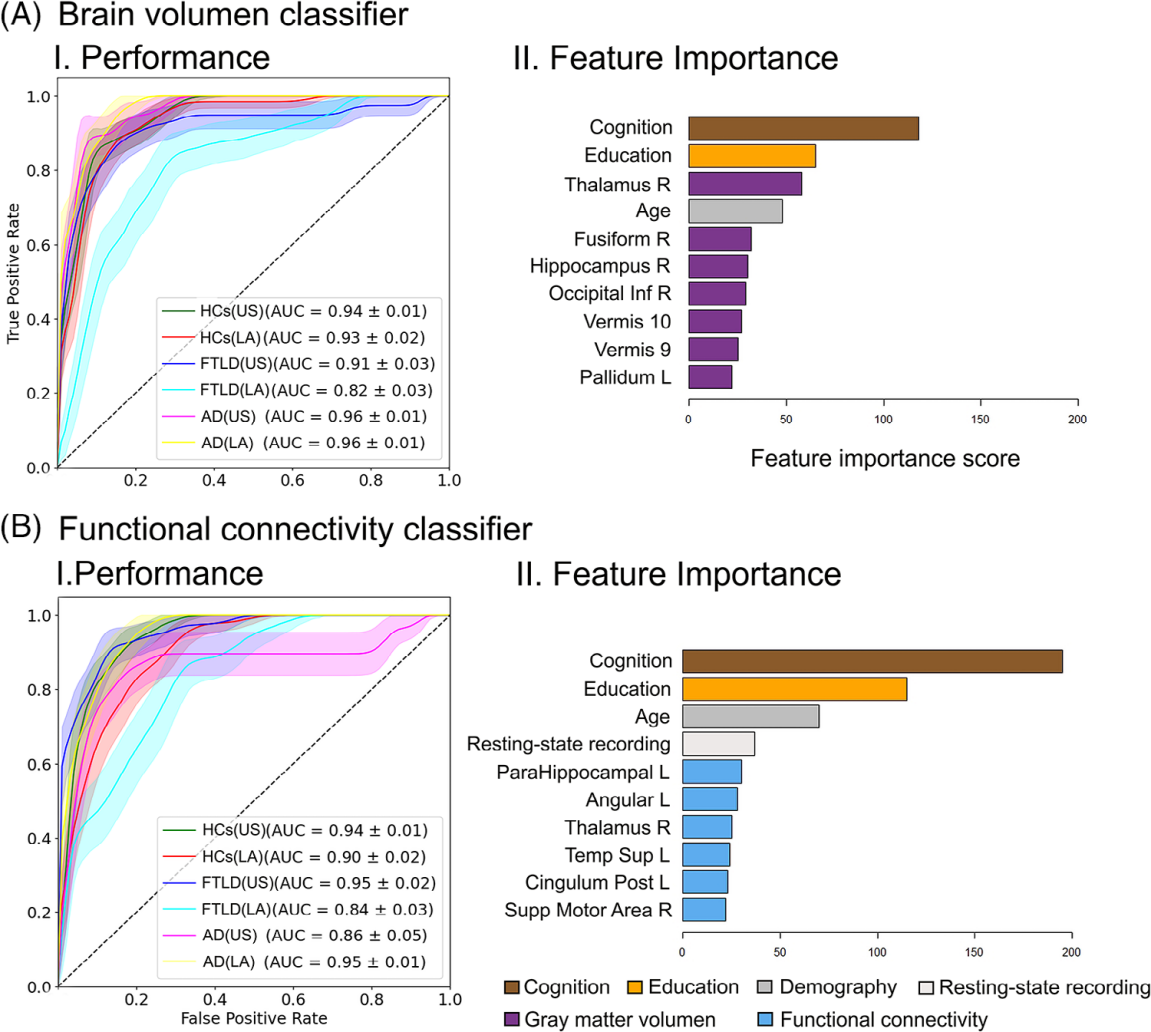
Multiclass classification across conditions and geographical regions. Classifiers used gray matter volume (A) and functional connectivity (B) data. Panel I displays performance through the ROC curve and the AUC score for the classification of each group against the others. Panel II displays the top 10 features, ranked by importance. Legend footnote: AD, Alzheimer's disease; AUC, area under curve; FTLD, frontotemporal dementia with lobar degeneration; HCs, healthy controls; Inf, inferior; LA, Latin America; L, left; Post, posterior; R, right; Supp, Supplementary; US, United States.

We employed a multiclass classifier combining ROI's gray matter volume, education, age, sex, and cognition, utilizing *k*‐fold cross‐validation. The algorithm predicted the condition across geographical regions, with a mean AUC of 0.92 (confidence intervals (CIs) of 0.90–0.94). The performance for each group ranged between 0.82 and 0.96 AUC scores (Figure 5A, panel I). As a result, education emerged as the second top feature in importance, surpassed by cognition (Figure 5A, panel II). Similarly, the model based on functional connectivity data yielded a mean AUC of 0.91 (CIs through *k*‐fold 0.88–0.93), with a range of 0.84–0.95 AUC score for each group (Figure 5B, panel I). Again, education played a pivotal role, ranking highest after cognition (Figure 5B, panel II). Table 2 provides comprehensive details, including accuracy, sensitivity, specificity, precision, recall, and F1 metrics for each condition across geographical regions regarding data from the gray matter volume and connectivity classifiers. Additional top features for gray matter volume included the right thalamus, age, right fusiform, left hippocampus, inferior occipital, cerebellar, and subcortical areas (Figure 5A, panel II). In functional connectivity, other critical features of importance comprised age, type of resting‐state recording, left angular, right thalamus, left superior temporal, and several prefrontal areas (Figure 5B, panel II). Thus education is a strong contributor compared to measures of brain structure and function in classifying conditions and geographical regions.

**TABLE 2:** Performance metrics for each condition by region in a multiclass classification using brain volume and functional connectivity data.

Group	Accuracy	Sensitivity	Specificity	Precision	Recall	F1
Brain volume data						
HCs (US)	0.94 ± 0.02	0.95 ± 0.02	0.93 ± 0.01	0.95 ± 0.02	0.84 ± 0.02	0.89 ± 0.03
HCs (LA)	0.94 ± 0.01	0.93 ± 0.01	0.95 ± 0.02	0.93 ± 0.01	0.89 ± 0.03	0.91 ± 0.02
FTLD (US)	0.91 ± 0.03	0.92 ± 0.01	0.89 ± 0.03	0.92 ± 0.01	0.92 ± 0.01	0.92 ± 0.01
FTLD (LA)	0.94 ± 0.02	0.95 ± 0.02	0.93 ± 0.01	0.95 ± 0.02	0.93 ± 0.02	0.94 ± 0.02
AD (US)	0.88 ± 0.01	0.92 ± 0.01	0.84 ± 0.02	0.92 ± 0.01	0.84 ± 0.02	0.89 ± 0.01
AD (LA)	0.92 ± 0.03	0.93 ± 0.01	0.92 ± 0.01	0.93 ± 0.01	0.89 ± 0.03	0.91 ± 0.03
Functional connectivity data						
HCs (US)	0.88 ± 0.03	0.84 ± 0.02	0.92 ± 0.01	0.84 ± 0.02	0.95 ± 0.02	0.90 ± 0.02
HCs (LA)	0.88 ± 0.03	0.84 ± 0.02	0.92 ± 0.01	0.84 ± 0.02	0.93 ± 0.01	0.89 ± 0.02
FTLD (US)	0.90 ± 0.02	0.92 ± 0.03	0.88 ± 0.03	0.92 ± 0.03	0.84 ± 0.02	0.88 ± 0.03
FTLD (LA)	0.91 ± 0.03	0.89 ± 0.03	0.95 ± 0.02	0.89 ± 0.03	0.88 ± 0.03	0.88 ± 0.02
AD (US)	0.88 ± 0.03	0.88 ± 0.03	0.89 ± 0.03	0.88 ± 0.03	0.92 ± 0.01	0.90 ± 0.01
AD (LA)	0.91 ± 0.02	0.95 ± 0.02	0.88 ± 0.03	0.95 ± 0.02	0.96 ± 0.02	0.95 ± 0.01

*Note*: Data are mean ± standard error.

Abbreviations: AD, Alzheimer's disease; FTLD, frontotemporal lobar degeneration; HCs, healthy controls; LA, Latin American; US, United States.

## 4 DISCUSSION

We investigated the impact of educational disparities on brain structure and functions in the context of healthy aging and dementia diversity, focusing on AD and FTLD across different geographical regions (LA and US). Our findings revealed that fewer years of education attainment were associated with reduced gray matter volume and lower functional connectivity of key brain areas. Notably, the LA cohort exhibited a lower educational level than the US, and these disparities played a crucial role in shaping the geographical differences in gray matter volume and connectivity. This factor explained 24.6%–98.7% of the geographical differences in brain structure and function, with a more pronounced impact on AD and FTLD. Finally, education emerged as the second top contributor in the multiclass classification across conditions (HCs, AD, and FTLD) and geographical regions (LA and the US). This finding highlights the critical role of education in explaining the geographical differences between LA and the US on healthy aging and dementia.

Although most previous reports have primarily evaluated cognitive reserve aspects,^13^, ^53^ our research assessed the impact of education on brain structure and function. The association of education with temporal areas^11^, ^54^ in our structural findings aligns with their role in long‐term memory,^55^ highlighting the potential impact of lifelong education on brain health. In addition, more years of education were associated with higher functional connectivity in relevant brain hubs, such as the orbitofrontal, posterior cingulate, and precuneus.^13^, ^14^, ^53^, ^54^ These areas are critical for integrating information,^56^ social cognition,^57^ and memory retrieval,^56^ all relevant processes for maintaining optimal brain health. Despite healthy aging, our findings indicate that education accounts for ≈24.6%–30% of the variance in brain structure and function observed between the LA and the US. To our knowledge, this is the first study showing how education influences brain structure and functional connectivity in participants from different backgrounds, including diverse LA populations and the US.

Education impacted brain structure and function, particularly in dementia, explaining 25.0%–98.7% of the geographical differences in these conditions. Specifically, brain areas susceptible to neurodegeneration, such as the temporal pole, orbitofrontal, and occipitoparietal areas,^58^, ^59^ showed selective educational associations. Brain differences between LA and the US across the three conditions diminished when controlling for education, especially in dementia. These results provide evidence for theoretical claims suggesting that education may contribute to the phenotypical heterogeneity of dementia in LA.^24^, ^25^, ^26^ In AD, geographical differences explained by education represented between 65.0% and 98.7% of the grey mattern and connectivity differences, respectively. This high percentage confirmed the more significant impact of educational disparities in AD than other neurodegenerative conditions.^9^, ^15^, ^60^ Notably, a considerable portion of these differences were in the temporal lobe, an area implicated in the pathogenesis of the disease.^28^, ^59^ In FTLD, education explained 25.0%–42.4% of the geographical differences, primarily encompassing the insula, superior temporal gyrus, and precuneus, areas usually impaired by this disorder.^58^ From a perspective of biological embedding,^61^ the structural inequality associated with low education can significantly influence different pathophysiological pathways, leading to reduced brain volume and connectivity. Physical and social exposomes in socioeconomically vulnerable populations, in which education provides an indirect measure, can influence whole‐body and brain inflammation, epigenetics, and allostatic load mechanisms.^62^ These factors start early in the development of LA populations.^63^ Conversely, cognitive stimulation and learning promote neuroplasticity and can increase brain reserve.^64^ This process helps to strengthen neural connections, which can enhance cognitive functions and potentially delay the development of neurodegenerative diseases. Our results underscore the disease‐specific nature of the effect of education on dementia subtypes and their variation across geographic regions.

Education resulted in the second top predictor discriminating across conditions and geographical regions in a multiclass classification framework. In clinical settings, a single neurodegenerative condition must be differentiated from multiple outcomes.^51^ The same approach was followed for our multiclass classifiers. In them, education was a relevant predictive feature across conditions (HCs, AD, FTLD) and geographical regions (LA, US). Cognition was the first top contributor, which is expected,^39^, ^51^ as it covaries with clinical diagnosis. By comparing each group against all others, the multiclass characterization always involved comparisons of patients and HCs, where cognitive differences are prominent. Our result showed that these factors (education and cognition) are more noticeable than measures of brain structure and function in discriminating among conditions, supporting recent reports of weaker brain–phenotype associations in more diverse dementia populations.^39^, ^51^ Including education may allow a higher performance on machine learning algorithms to classify dementia.^51^, ^65^ Together, these results suggest that multimodal dementia characterization, including socioeconomic factors (education), can provide more robust discrimination of brain health and disease across global samples.

Multiple methodological steps were performed to control potential confounders. Effects were robust even when controlled for age, sex, TIV, and type of resting‐state recording. Imaging quality and inter‐scan variability were also controlled in the results. Nonetheless, essential limitations call for further research. Although we lack direct measures of socioeconomic status, education represents the more useful metric to register this component^2^; future studies should consider other measures such as income and profession.^2^ Years of education attainment may be a rough estimate of the quality of education, especially regarding multi‐country studies. Despite excluding the Latino population, the demographic landscape of the US remains complex due to the interplay of socio‐ethnic and economic factors.^66^ However, our work suggests that the variability in brin measures is lower in LA than in the US. Indeed, one year of schooling in the US may be equivalent to three or more years in LA.^67^ LA exhibits vast cultural and economic diversity.^19^, ^34^ Our research demonstrates that, despite this heterogeneity, the US consistently displays larger educational correlates of brain structure and connectivity than each LA country (Supplementary Material 5). Future investigations that integrate cultural and socioeconomic variables could provide valuable insights into how specific local contexts influence the association between education and brain health. Future efforts should develop metrics that harmonize education across different countries while integrating digital literacy. Although our study does not directly investigate the impact of education on cognitive performance, we accounted for cognitive differences. Specifically, we found no significant variations in cognition among HCs, AD, and FTLD between the LA and US groups. Assessing the role of educational disparities in clinical severity, functional abilities, and specific symptomatology across AD and FTLD may also expand the relevance of current results. Future studies in Latino and other diverse populations should also disentangle the effects of race/ethnicity from geographical origins.

In conclusion, our results reveal an important influence of educational disparities on brain health and dementia across LA and the US. Correlates of education presented a greater performance in US participants than in their LA counterparts. Furthermore, education explained a substantial part of the geographical differences in gray matter volume and connectivity and was crucial in classifying conditions. These findings emphasize the need to integrate individual educational attainment measures in approaches to prevent, diagnose, and intervene in neurodegenerative disorders.^24^, ^25^, ^26^ Our study represents a significant effort in integrating data from different regions following the ADNI protocols, opening new opportunities to replicate this approach with data from other parts of the world. As we move forward, it becomes imperative to incorporate educational factors into tailored models of brain health, offering a more comprehensive understanding of the complexities involved in neurodegenerative conditions.

## CONFLICT OF INTEREST STATEMENT

Raul Gonzalez‐Gomez, Agustina Legaz, Sebastián Moguilner, Josephine Cruzat, Hernán Hernández, Sandra Baez, Rafael Cocchi, Carlos Coronel‐Olivero, Vicente Medel, Enzo Tagliazuchi, Joaquín Migeot, Carolina Ochoa‐Rosales, Marcelo Adrián Maito, Pablo Reyes, Hernando Santamaria Garcia, Maria E. Godoy, Shireen Javandel, Diana L. Matallana, José Alberto Avila‐Funes, Martín A. Bruno, Juan F. Cardona, Ignacio L. Brusco, Martín A. Bruno, Ana L. Sosa Ortiz, Stefanie D. Pina‐Escudero, Leonel T. Takada, Katherine L. Possin, Maira Okada de Oliveira, Francisco Lopera, Brian Lawlor, Bruce Miller, Jennifer S. Yokoyama, Cecilia Gonzalez are not declaring any conflicts of interest. Adolfo M. García is supported with funding from the National Institute on Aging of the National Institutes of Health (NIH): R01AG075775); Agencia Nacional de Investigacion y desarrollo de Chile/Fondo Nacional de Desarrollo Cientifico y Tecnologico (ANID/FONDECYT)  Regular:210176, 1210195; and Programa Interdisciplinario de Investigación Experimental en Comunicación y Cognición (PIIECC), Facultad de Humanidades, Universidad de Santiago de Chile. Andrea Slachevsky is partially supported by ANID/FONDECYT Regular: 1231839 and ANID/FONDAP 15150012. Nilton Custodio is partially supported by NIH: AG057234, R56AG069118‐01, SG‐21‐715176‐LATAM FINGERS, 24AARG‐D‐1246942. Elisa de Paula França Resende is partially supported by grants from the NIH: 1R21AG069252‐01 and Rainwater foundation grant. Victor Valcour is partially supported by grants from the NIH, Bluefield Consortium and Alzheimer's Association. Kun Hu is partially supported by grants from the NIH: RF1AG059867, RF1AG064312, and R01AG083799. Agustin Ibanez is supported by grants from the Multi‐Partner Consortium to Expand Dementia Research in Latin America (ReDLat), Fogarty International Center (FIC), NIH: R01 AG057234, R01 AG075775, R01 AG21051, R01 AG083799, CARDS‐NIH), Alzheimer's Association (SG‐20‐725707), Rainwater Charitable Foundation—The Bluefield project to cure FTD, and Global Brain Health Institute, ANID/FONDECYT Regular (1210195, 1210176 and 1220995); and ANID/FONDAP/15150012. The contents of this publication are solely the author's responsibility and do not represent the official views of these institutions. Author disclosures are available in the supporting information.

## CONSENT STATEMENT

All human subjects provided a written informed consent. Alzheimer's Disease Neuroimaging Initiative (ADNI) and Neuroimaging in Frontotemporal Dementia (NIFD) data sets are available in their online repository (https://ida.loni.usc.edu). For Multi‐Partner Consortium to Expand Dementia Research in Latin America (ReDLat) data, specific research projects can be submitted to the board for approval of a data‐sharing agreement (https://red‐lat.com). The code for this study's data analysis is available from the corresponding author upon reasonable request.

## Supporting information

Supporting Information

Supporting Information
