# Supplementary material for: Educational disparities in brain health and dementia across Latin America and the United States
Source: Alzheimers Dement. 2024 Aug 13;20(9):5912–25. doi: 10.1002/alz.14085 (PMC11497666; doi:10.1002/alz.14085)
Supplement: Supplementary file 1 — Supporting Information [file ALZ-20-5912-s001.docx]

**Supplementary Files**

**Supplementary Material 1:** Atrophy in AD and FTLD

**Supplementary Material 2:** Details of demography and cognition across conditions, geographical region, and dataset

**Supplementary Material 3:** Inter-scan variability

**Supplementary Material 4:** T1-weighted and resting-state functional MRI quality.

**Supplementary Material 5:** Intercultural differences

**Supplementary Material 6:** Cognition impairment in patients

**Supplementary Material 7:** Significant clusters in voxel-based morphometry and functional connectivity analysis

**Supplementary Material 1:** Atrophy in AD and FTLD

The patients included in this study exhibit characteristic atrophy patterns (**Supplementary Figure 1A**, panel I). This pattern is consistent across patients from LA (**Supplementary Figure 1A**, panel II) and US (**Supplementary Figure 1A**, panel III). Specifically, all patients diagnosed with AD display atrophy in regions encompassing the hippocampus, temporal lobe, occipital areas, and the parietal and prefrontal cortical hubs (**Supplementary Figure 1B**, panel I). This established pattern is consistent among AD patients in the US (**Supplementary Figure 1B**, panel III) but exhibits a reduction among patients from LA (**Supplementary Figure 1B**, panel II). In the case of patients diagnosed with FTLD, pronounced atrophy is observed in the prefrontal and temporal cortices, with concomitant damage observed in the orbitofrontal, insular, and anterior-temporal regions (**Supplementary Figure 1C**, panel I). This identified pattern is also evident among FTLD patients in the US (**Supplementary Figure 1C**, panel III); however, a reduction is observed in LA (**Supplementary Figure 1C**, panel II).


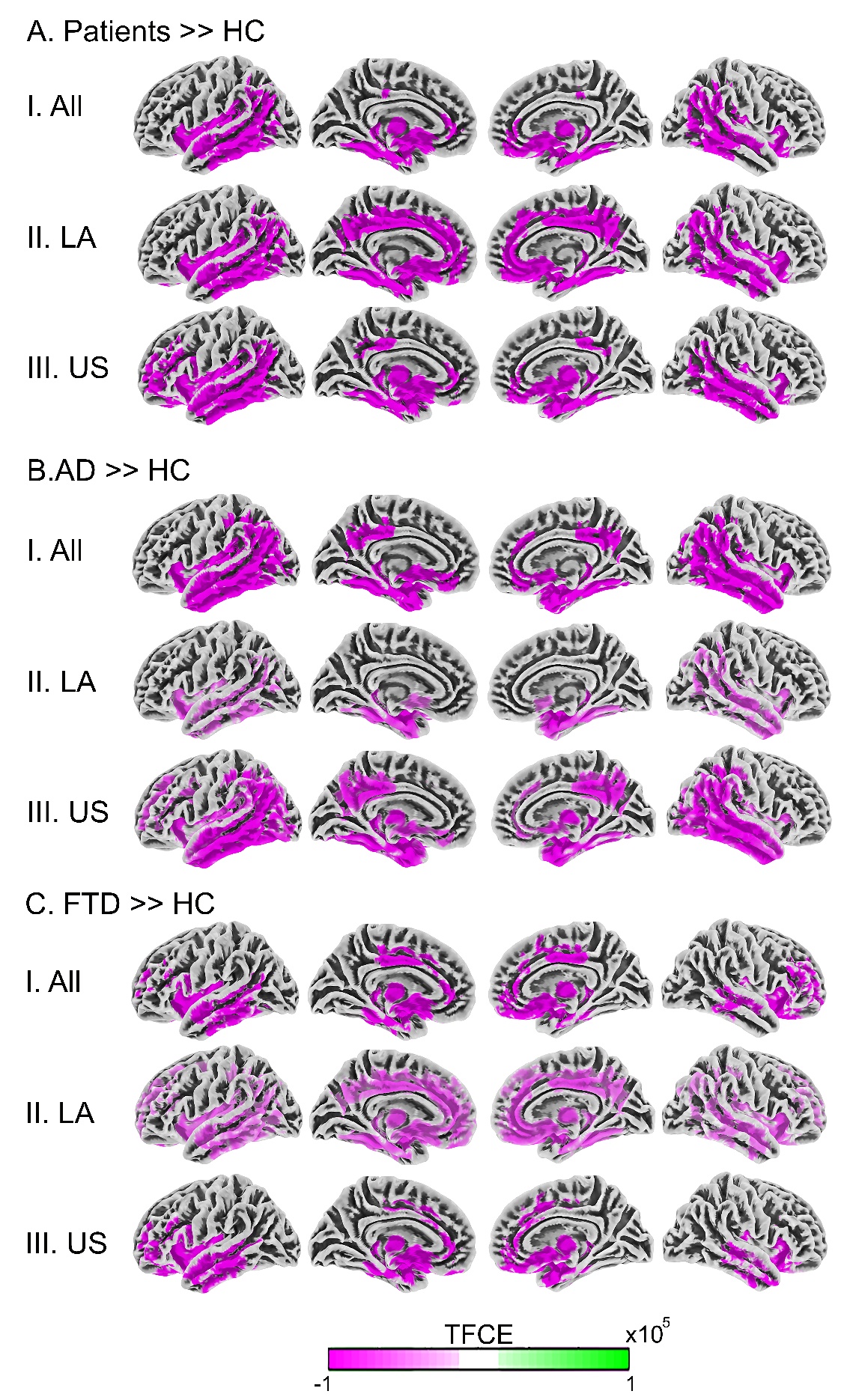
**Supplementary Figure 1.** Atrophy patterns in neurodegenerative patients (A), stratified by clinical diagnosis of Alzheimer's disease (B) and Fronto^1,2^temporal lobar degeneration (C). The analysis was conducted with the entire subject cohort (Panel I) and separately for two geographical regions, Latin America (Panel II) and the United States (Panel III). All analyses were executed utilizing voxel-based morphometry adjusting for age, sex, and total intracranial volume. Moreover, multiple comparisons were corrected using the threshold-free cluster enhancement (TFCE) method to address family-wise errors. Legend footnote: LA, Latin America; US, United States.

**Supplementary Material 2:** Details of demography and cognition across conditions, geographical regions, and dataset

**Supplementary Table 1.** **Demography of subjects in the T1 and rs-fMRI datasets**

|  |  |  | Age | | | | Sex | | Education | | | | Cognition (MMSE) | | | |
| --- | --- | --- | --- | --- | --- | --- | --- | --- | --- | --- | --- | --- | --- | --- | --- | --- |
|  |  | n | mean | SD | miss | stats | Fem | Stats | mean | SD | miss | stats | mean | SD | miss | stats |
| T1 | | | | | | | | | | | | | | | | |
| HC | LA | 318 | 64.6 | 11 | 2 | *Χ*^2^=29.6 | 205 | *Χ*^2^=3.3 | 15.1 | 4.2 | 8 | *Χ*^2^=33.4 | 28.6 | 1.6 | 15 | *Χ*^2^=0.7 |
|  | US | 307 | 69.5 | 7.5 |  | p<.001 | 175 | p=0.07 | 17.2 | 5.3 | 15 | p<.001 | 28.1 | 2.9 | 5 | p=0.41 |
| AD | LA | 193 | 72.2 | 7.8 |  | *Χ*^2^=29.1 | 129 | *Χ*^2^=5.0 | 10.5 | 5.1 |  | *Χ*^2^=132 | 18.7 | 3.6 | 8 | *Χ*^2^=0.75 |
|  | US | 192 | 67.2 | 9.2 |  | p<.001 | 106 | p=0.03 | 16.3 | 2.5 |  | p<.001 | 17.8 | 6.4 | 22 | p=0.39 |
| FTDL | LA | 208 | 65.8 | 9.7 | 4 | *Χ*^2^=7.8 | 102 | *Χ*^2^=2.8 | 12.9 | 5.2 | 3 | *Χ*^2^=52.4 | 22.3 | 5.5 | 8 | *Χ*^2^=0.1 |
|  | US | 194 | 63.7 | 7.3 |  | p<.01 | 78 | p=0.09 | 16.3 | 2.9 | 16 | p<.001 | 22.5 | 5.7 | 15 | p=0.75 |
| rs-fMRI | | | | | | | | | | | | | | | | |
| HC | LA | 240 | 67.5 | 9.5 | 7 | *Χ*^2^=57.4 | 159 | *Χ*^2^=6.4 | 15.4 | 4.5 | 3 | *Χ*^2^=13.9 | 28.7 | 1.6 | 16 | *Χ*^2^=2.6 |
|  | US | 332 | 62.8 | 12 | 2 | p<.001 | 184 | p=0.01 | 16.9 | 2.3 | 17 | p<.001 | 27.9 | 2.6 | 5 | p=0.42 |
| AD | LA | 168 | 72.4 | 7.6 | 1 | *Χ*^2^=17.8 | 114 | *Χ*^2^=10.6 | 10.9 | 5 | 2 | *Χ*^2^=101 | 20.4 | 2.8 | 7 | *Χ*^2^=2.4 |
|  | US | 152 | 67.9 | 9.4 |  | p<.001 | 75 | p<0.01 | 16.3 | 2.2 | 6 | p<.001 | 19.9 | 4.2 | 15 | p=0.12 |
| FTDL | LA | 177 | 66.2 | 8.7 | 4 | *Χ*^2^=8.7 | 87 | *Χ*^2^=3.0 | 12.7 | 5.2 | 4 | *Χ*^2^=48.7 | 22.3 | 5.5 | 9 | *Χ*^2^=1.1 |
|  | US | 194 | 63.7 | 7.3 |  | p=.003 | 77 | p=0.08 | 16.2 | 2.8 | 15 | p<.001 | 22.6 | 5.7 | 15 | p=0.97 |

A comparison between regions (LA vs. US) was performed for each condition. Kruskal-Wallis test was used to compare continuous variables, as the data was non-normally distributed (Shapiro-Will test p<0.001). The *Χ*^2^ test for equality of proportions with continuity correction was used to compare sex variables. All p values were adjusted using Bonferroni correction and set to p<0.05. Legend footnote: *Χ*^2^, chi-squared; AD, Alzheimer’s disease; fem, females; FTLD, Frontotemporal lobar degeneration; HC, healthy controls; LA, Latin American countries; miss, missing data; MMSE, Mini-Mental State Examination; US, United States of America; SD, standard deviation.

**Supplementary Material 3:** Inter-scan variability

**Supplementary Tables 2** and **3** presented the frequencies of MRI scanner models for acquiring T1-weighted and resting-state functional MRI data, respectively. **Supplementary Figures 2** and **3** showed that dummy variables codifying the model of the scanner appeared at the bottom of the features' importance ranks.

**Supplementary Table 2**. MRI scanner models were employed for recording T1-weighted images of the study groups.

|  | Model | AD_LA | AD_US | FTLD_LA | FTLD_US | HC_LA | HC_US |
| --- | --- | --- | --- | --- | --- | --- | --- |
| Scanner 1 | GE DISCOVERY | 0 | 16 | 0 | 0 | 0 | 17 |
| Scanner 2 | GE Signa Voyager | 0 | 4 | 0 | 0 | 0 | 5 |
| Scanner 3 | GE Signa Premier | 14 | 0 | 12 | 0 | 23 | 1 |
| Scanner 4 | Philips Achieva | 47 | 14 | 130 | 0 | 166 | 8 |
| Scanner 5 | Philips Ingenia | 22 | 5 | 28 | 0 | 39 | 6 |
| Scanner 6 | Philips Intera | 22 | 0 | 0 | 0 | 32 | 0 |
| Scanner 7 | Philips Prodiva | 0 | 0 | 0 | 0 | 1 | 0 |
| Scanner 8 | Siemens Aera | 5 | 0 | 0 | 0 | 0 | 0 |
| Scanner 9 | Siemens Avanto | 4 | 0 | 13 | 0 | 0 | 0 |
| Scanner 10 | Siemens Biograph | 0 | 1 | 0 | 0 | 0 | 0 |
| Scanner 11 | Siemens Prisma | 0 | 3 | 0 | 0 | 0 | 13 |
| Scanner 12 | Siemens Prisma fit | 0 | 73 | 0 | 71 | 2 | 60 |
| Scanner 13 | Siemens Skyra | 67 | 0 | 24 | 0 | 37 | 0 |
| Scanner 14 | Siemens Symphony | 10 | 0 | 1 | 0 | 16 | 0 |
| Scanner 15 | Siemens TrioTim | 0 | 71 | 0 | 123 | 0 | 187 |
| Scanner 16 | Siemens Verio | 2 | 5 | 0 | 0 | 2 | 10 |

**Supplementary Table 3**. MRI scanner models were employed to record the study groups' resting functional MRIs.

|  | Modelo | AD_LA | AD_US | FTLD_LA | FTLD_US | HC_LA | HC_US |
| --- | --- | --- | --- | --- | --- | --- | --- |
| Scanner 1 | GE DISCOVERY | 0 | 16 | 0 | 0 | 0 | 25 |
| Scanner 2 | GE Signa Voyager | 0 | 4 | 0 | 0 | 0 | 5 |
| Scanner 3 | GE Signa Premier | 11 | 0 | 0 | 0 | 26 | 1 |
| Scanner 4 | Philips Prodiva | 0 | 0 | 0 | 0 | 1 | 0 |
| Scanner 5 | Philips Achieva | 55 | 14 | 122 | 0 | 149 | 8 |
| Scanner 6 | Philips Ingenia | 28 | 4 | 27 | 0 | 39 | 7 |
| Scanner 7 | Siemens Avanto | 8 | 0 | 15 | 0 | 0 | 0 |
| Scanner 8 | Siemens Prisma | 0 | 4 | 0 | 0 | 0 | 16 |
| Scanner 9 | Siemens Prisma fit | 0 | 54 | 0 | 70 | 0 | 68 |
| Scanner 10 | Siemens Skyra | 66 | 0 | 13 | 0 | 25 | 0 |
| Scanner 11 | Siemens TrioTim | 0 | 53 | 0 | 124 | 0 | 191 |
| Scanner 12 | Siemens Verio | 0 | 3 | 0 | 0 | 0 | 11 |

**
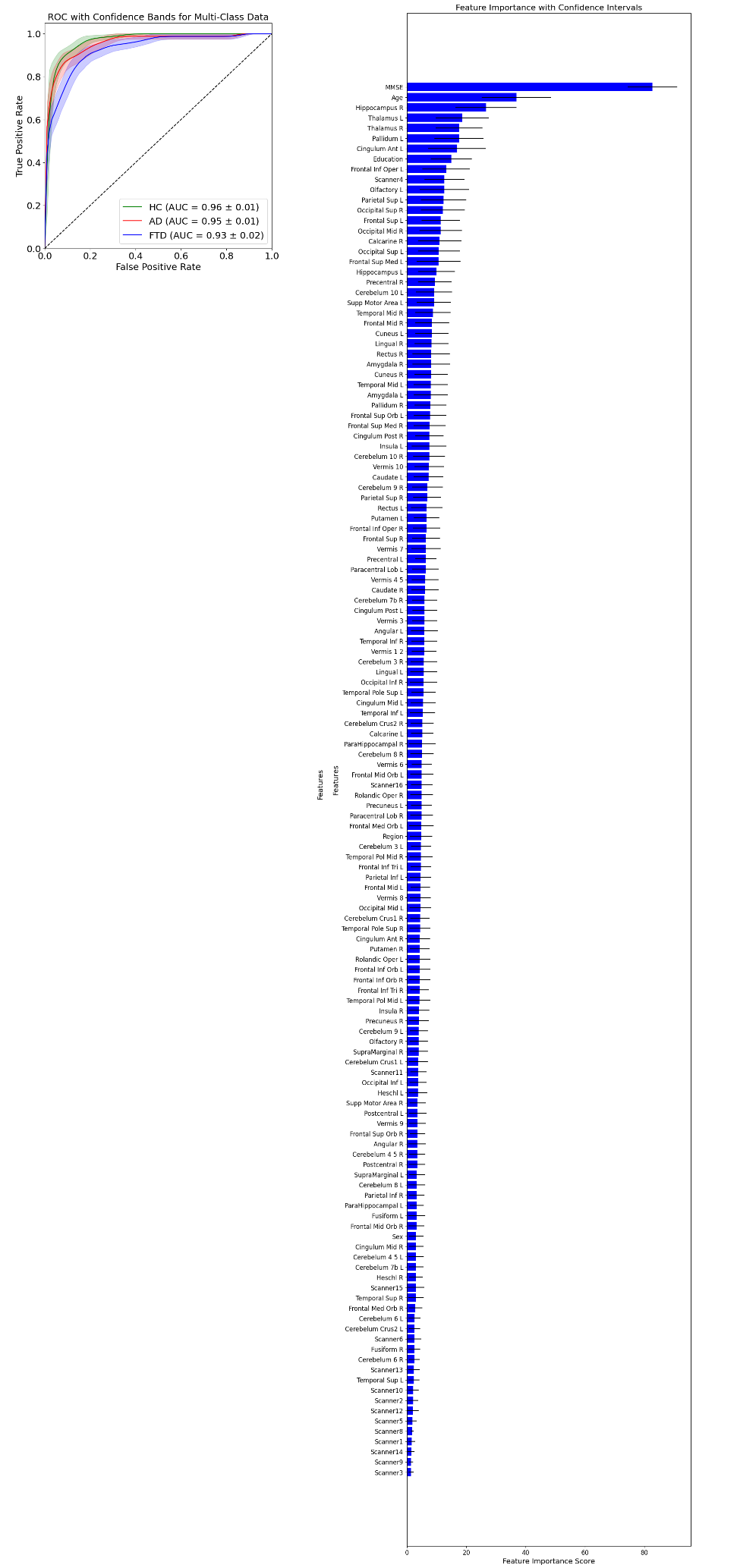
**

**Supplementary Figure 2. Multiclass classification of the condition using brain volume, demography, cognition, and education data.** The employed classifier followed a one-vs-rest strategy. Their performance depicts Receiver Operating Characteristic (ROC) curves and the corresponding Area Under the Curve (AUC) scores. Legend footnote: AD, Alzheimer's Disease; FTLD, frontotemporal lobar degeneration; HC, healthy controls.

**
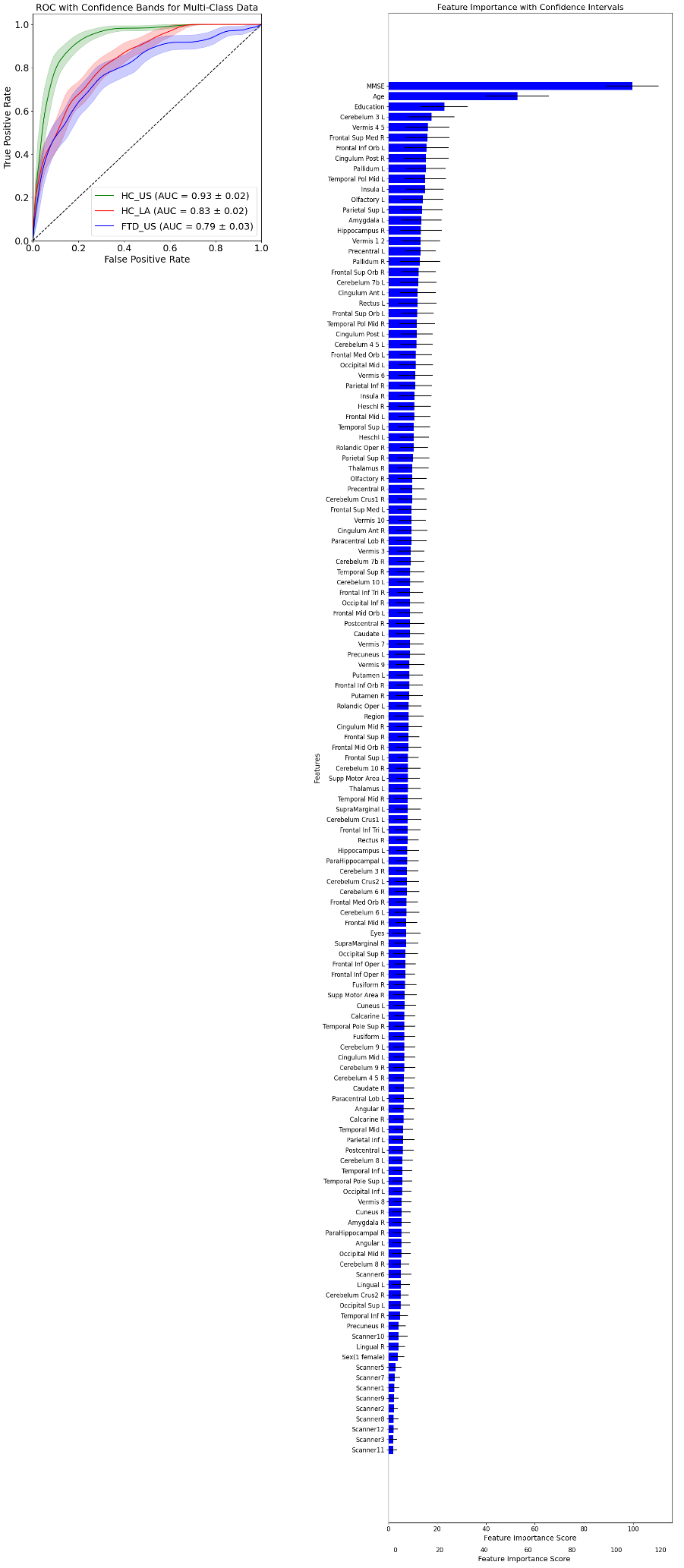
**

**Supplementary Figure 3. Multiclass classification of conditions using brain connectivity, demography, cognition, and education data.** The employed classifier followed a one-vs-rest strategy. Their performance is presented through Receiver Operating Characteristic (ROC) curves and the corresponding Area Under the Curve (AUC) scores. Legend footnote: AD, Alzheimer's Disease; FTLD, frontotemporal lobar degeneration; HC, healthy controls.

**Supplementary Material 4:** T1-weighted and resting-state functional MRI quality.

To extract no-reference image quality metrics from T1-weighted and resting-state functional MRI, the software MRIQC was used (<https://mriqc.readthedocs.io/>). This open-source software test for **reliability and robustness ^3^.** The no-reference metrics of this software are measurements of some aspect of the actual image, which cannot be compared to a reference value for the metric since there is no ground truth about what this number should be. **Nonetheless, it is feasible to analyze the distribution of these metrics and identify outliers within the dataset. We relied on two primary parameters for the T1-weighted MRI quality assessment (Supplementary Figure 6). Firstly, the coefficient of joint variation is associated with detecting pronounced head motion artifacts ^4^. Secondly, the contrast-to-noise ratio quantifies the distinctness between gray and white matter tissue distributions. ^5^. Furthermore, two metrics were employed to gauge the quality of resting-state functional MRI (Supplementary Figure 7). The first metric, signal-to-noise ratio, pertains to spatial information, while the second involves the standard deviation of the temporal derivative of time course variances over voxels ^6^. Notably, neither of these metrics revealed outliers in the dataset utilized, confirming the overall quality of the data obtained.**

**
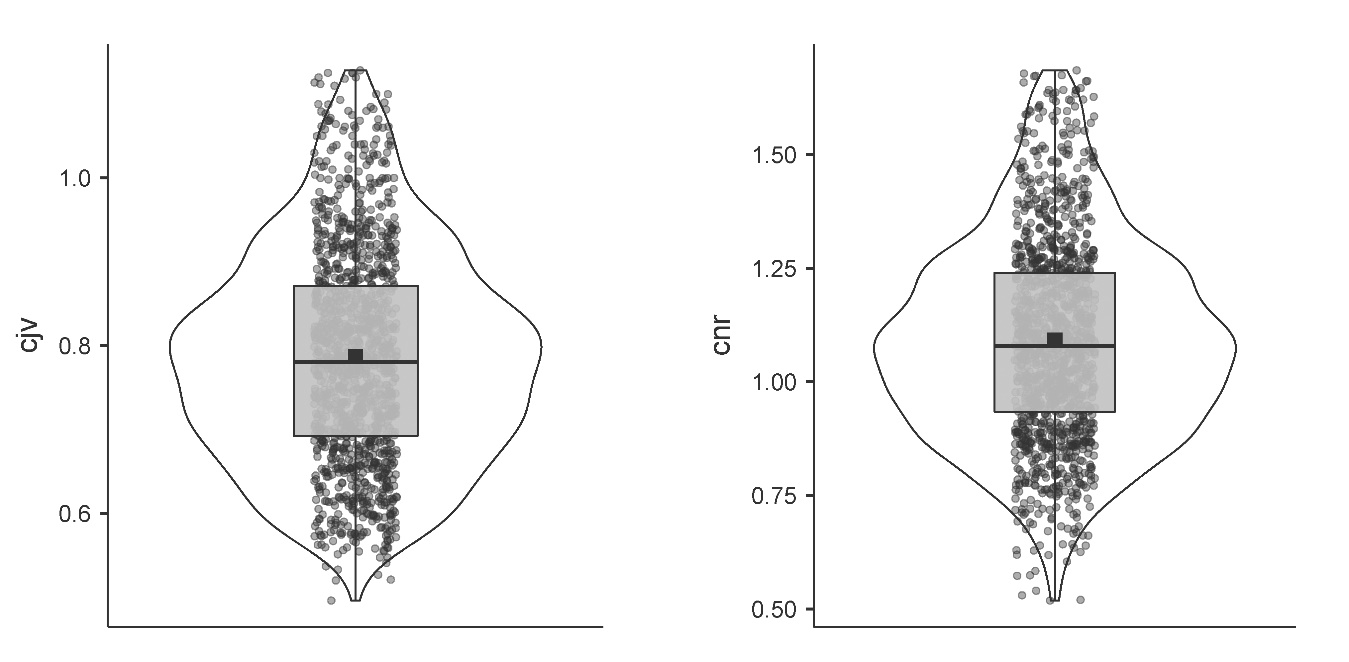
**

**Supplementary Figure 6.** No-reference image quality metrics from T1-weighted MRI **within the used dataset**. **The left panel illustrates the coefficient of joint variation (cjv), while the right panel displays the contrast-to-noise ratio (cnr).**

**
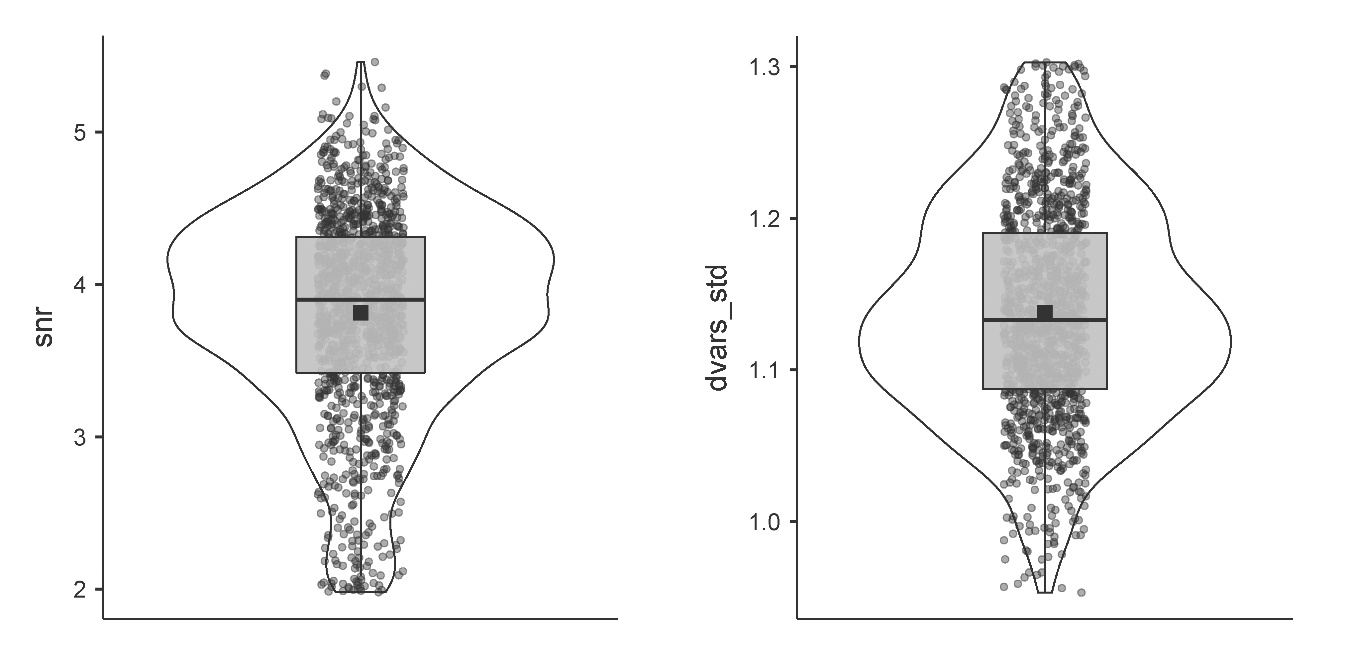
**

**Supplementary Figure 7.** No reference image quality metrics from resting-state functional MRI **within the used dataset. The left panel illustrates the signal-to-noise ratio (SNR), while the right panel displays the standard deviation of the temporal derivative of time course variances over voxels (dvars_std).**

**Supplementary Material 5:** Inter-cultural effects

We employed a Dunnett’s test adjusted for false discovery rate (FDR) to compare the educational correlates of each LA country vs. the US for every modality. **Supplementary Table 4** shows the demographics of each condition by country. Out of 19 possible comparisons, 89.5% indicate that the US population statistically exhibits higher educational correlates than each LA country (**Supplementary Table 5** and **Supplementary Figure 8**). Even in the two comparisons that were not statistically significant, there was still a noticeable trend towards larger correlates for the US.

**Supplementary Table 4**. Demographics of each condition by country

| **Country** | **Group** | **n** | **Average age** | **Std. Dev. of Age** | **No. female** |
| --- | --- | --- | --- | --- | --- |
| **T1** | | | | | |
| Argentina | AD | 28 | 72.12 | 9.15 | 17 |
| Argentina | HCs | 101 | 59.84 | 13.8 | 78 |
| Argentina | FTLD | 54 | 66.81 | 15.71 | 17 |
| Chile | AD | 56 | 74.68 | 7.61 | 32 |
| Chile | HCs | 82 | 72.73 | 5.72 | 59 |
| Chile | FTLD | 45 | 67.73 | 10.12 | 23 |
| Colombia | AD | 32 | 70.9 | 6.84 | 29 |
| Colombia | HCs | 88 | 61.91 | 7.73 | 45 |
| Colombia | FTLD | 110 | 64.93 | 7.78 | 62 |
| Mexico | AD | 35 | 70.94 | 7.57 | 24 |
| Mexico | HCs | 32 | 58 | 11.26 | 16 |
| Peru | AD | 41 | 72.25 | 8.27 | 27 |
| Peru | HCs | 15 | 70.93 | 9.65 | 7 |
| rs fMRI | | | | | |
| Argentina | AD | 28 | 75.5 | 6.04 | 20 |
| Argentina | HCs | 76 | 65.09 | 9.82 | 57 |
| Argentina | FTLD | 38 | 68.48 | 10.89 | 18 |
| Chile | AD | 98 | 72.7 | 7.86 | 65 |
| Chile | HCs | 78 | 61.28 | 16.9 | 56 |
| Chile | FTLD | 42 | 66.81 | 9.08 | 13 |
| Colombia | AD | 42 | 69.42 | 7 | 29 |
| Colombia | HCs | 86 | 61.91 | 7.77 | 46 |
| Colombia | FTLD | 97 | 65.15 | 7.68 | 56 |

**Supplementary Table 5**. Statistics of Dunnett’s test, adjusted for false discovery rate (FDR), to compare the educational correlates of each Latin American country vs. the US subjects for every modality.

| **Group** | **Comparison** | **Z** | **P_unadj** | **P_adj (FDR)** |
| --- | --- | --- | --- | --- |
| **Structural correlates** | | | | |
| HCs | Argentina - US | -0.8527 | 0.394 | 0.422 |
|  | Chile - US | -7.431 | 1.08E-13 | 1.62E-12 |
|  | Colombia - US | -6.1887 | 6.06E-10 | 4.55E-09 |
|  | Mexico - US | -2.2465 | 0.0247 | 0.0529 |
|  | Peru - US | -3.897 | 9.74E-05 | 0.000292 |
| AD | Argentina - US | -2.8227 | 0.00476 | 0.00893 |
|  | Chile - US | -9.0653 | 1.24E-19 | 1.86E-18 |
|  | Colombia - US | -3.7993 | 0.000145 | 0.000435 |
|  | Mexico - US | -3.8314 | 0.000127 | 0.000478 |
|  | Peru - US | -3.9823 | 6.83E-05 | 0.000341 |
| FTLD | Argentina - US | -2.7808 | 0.00542 | 0.0271 |
|  | Chile - US | -4.3412 | 1.42E-05 | 0.000106 |
|  | Colombia - US | -7.3681 | 1.73E-13 | 2.60E-12 |
| **Functional correlates** | | | | |
| HCs | Argentina - US | -5.45438 | 4.91E-08 | 2.95E-07 |
|  | Chile - US | -4.68866 | 2.75E-06 | 1.65E-05 |
|  | Colombia - US | -5.73713 | 9.63E-09 | 5.78E-08 |
| AD | Argentina - US | -4.87273 | 1.10E-06 | 6.60E-06 |
|  | Chile - US | -5.98921 | 2.11E-09 | 1.27E-08 |
|  | Colombia - US | -6.81764 | 9.25E-12 | 5.55E-11 |
| FTLD | Argentina - US | -4.33674 | 1.45E-05 | 8.68E-05 |
|  | Chile - US | -5.70086 | 1.19E-08 | 7.15E-08 |
|  | Colombia - US | -8.19807 | 2.44E-16 | 1.33E-15 |


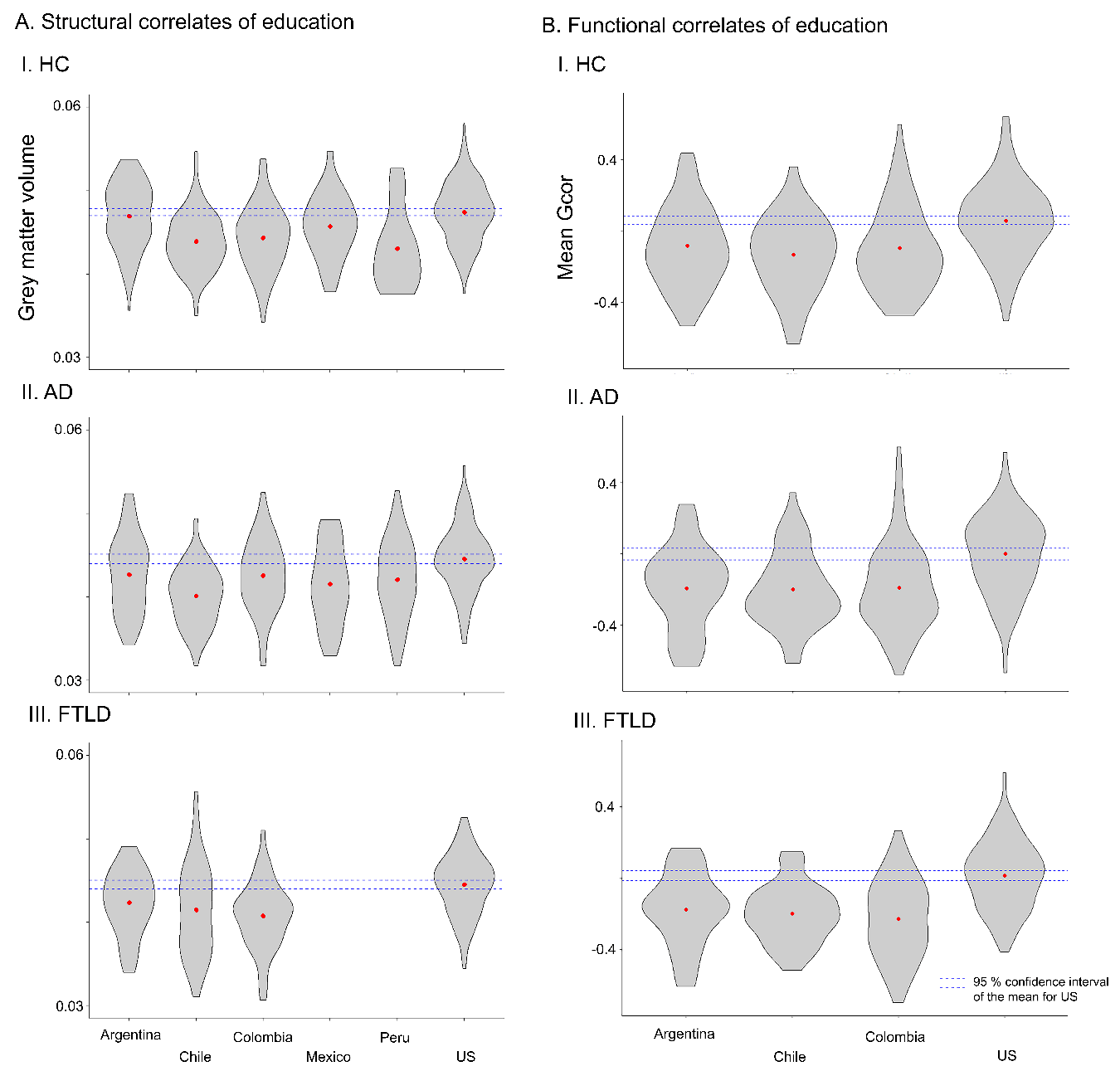


**Supplementary Figure 8:** Graphical representation of the comparisons of structural (A) and functional (B) correlates of education for each Latin American country vs. the US subjects. AD, Alzheimer's disease; FTLD, frontotemporal lobar degeneration; GCOR, global correlation; HCs, healthy controls.

**Supplementary Material 6:** Cognitive impairment in patients

As expected, MMSE scores were significantly different when comparing AD and FTD groups against the HC group in both regions and datasets (**Supplementary Table 6**).

**Supplementary Table 6.** Statistical differences in MMSE score in AD and FTLD patients vs. healthy controls in T1 and rs-fMRI datasets.

|  | **LA** | **US** |
| --- | --- | --- |
|  | **T1** | |
| **AD** | *Χ*^2^=29.4, p<.001 | *Χ*^2^=23.2, p<.001 |
| **FTLD** | *Χ*^2^=21.4, p<.001 | *Χ*^2^=18.1, p<.001 |
|  | **rs-fMRI** | |
| **AD** | *Χ*^2^=24.0, p<.001 | *Χ*^2^=22.8, p<.001 |
| **FTLD** | *Χ*^2^=19.7, p<.001 | *Χ*^2^=18.0, p<.001 |

As the Shapiro-Will test showed p<0.001 for the MMSE variable, we used the Kruskal-Wallis test for the comparisons. All p values were adjusted using Bonferroni correction and set to p<0.05. *Χ*^2^: chi-squared from Kruskall Wallis test, AD: Alzheimer’s disease, FTDL: Frontotemporal dementia lobar degeneration, HC: healthy controls, LA: Latin American countries, MMSE: Mini-Mental State Examination, US: United States.

**Supplementary Material 7:** Significant clusters in voxel-based morphometry and functional connectivity analysis

Supplementary Tables 7 and 8 present details from the results of voxel-based morphometry and functional connectivity analysis, respectively**.**

**Supplementary Table 7:** Significant clusters in voxel-based morphometry analisis.

| Cluster (x,y,z) | | | ROI | size (# of voxels) | peak TFCE | peak p-FWE | peak p-FDR | peak p-unc |
| --- | --- | --- | --- | --- | --- | --- | --- | --- |
| Functional correlates of Education | | | | | | | | |
| -33 | -75 | -57 | Temporal Cortex | 153476 | 11291 | <0.001 | <0.001 | <0.001 |
| -25.5 | 63 | -15 | Frontal Superior | 547 | 291 | <0.001 | <0.001 | <0.001 |
| HCs: geographical comparisons overall | | | | | | | | |
| 19.5 | 4.5 | -39 | Cerebellum Right | 70770 | -53273 | <0.001 | <0.001 | <0.001 |
| 6 | 34.5 | -31.5 | Frontal Superior | 1363 | -23856 | <0.001 | <0.001 | <0.001 |
| HCs: geographical comparisons corrected through education | | | | | | | | |
| 19.5 | 1.5 | -40.5 | Temporal Pole Rigth | 59782 | -47177 | <0.001 | <0.001 | <0.001 |
| 6 | 34.5 | -31.5 | Orbitofrantal Gyrus | 444 | -19660 | <0.001 | <0.001 | <0.001 |
| 4.5 | -19.5 | 7.5 | Sub -lobar | 4289 | -53517 | <0.001 | <0.001 | <0.001 |
| AD: geographical comparisons overall | | | | | | | | |
| -18 | -55 | -50 | Cerebellum Left | 54040 | -14404 | <0.001 | <0.001 | <0.001 |
| -54 | -7.5 | 47 | Precentral Left | 2519 | -7583 | <0.001 | <0.001 | <0.001 |
| 5 | -7.5 | -1.5 | Brain stem | 1281 | -8142 | <0.001 | <0.001 | <0.001 |
| AD: geographical comparisons corrected through education | | | | | | | | |
| 12 | -61.5 | -61.5 | Cerebellum Right | 9022 | -8854 | <0.001 | <0.001 | <0.001 |
| 21 | -10 | -34 | Temporal Lobe Right | 5005 | -7532 | <0.001 | <0.001 | <0.001 |
| -21 | -12 | -33 | Temporal Lobe Left | 606 | -7294 | <0.001 | <0.001 | <0.001 |
| FTLD: geographical comparisons overall | | | | | | | | |
| 3 | -80 | -4.5 | Lingual Right | 104539 | -42798 | <0.001 | <0.001 | <0.001 |
| FTLD: geographical comparisons corrected through education | | | | | | | | |
| 1.5 | -63 | 10.5 | Cerebellum Right | 93089 | -33513 | <0.001 | <0.001 | <0.001 |

**Supplementary Table 8:** Significant clusters in functional connectivity analisis.

| Cluster (x,y,z) | | | representative ROI | size (# of voxels) | peak TFCE | peak p-FWE | peak p-FDR | peak p-unc |
| --- | --- | --- | --- | --- | --- | --- | --- | --- |
| Functional correlates of Education | | | | | | | | |
| -14 | -30 | -4 | Cingulum posterior | 3355 | 1252.99 | <0.001 | <0.001 | <0.001 |
| 36 | 58 | -2 | Frontal Cortex | 1743 | 818.52 | 0.008 | 0.001786 | <0.001 |
| 60 | 0 | 6 | Insula Right | 284 | 713.96 | 0.026 | 0.003496 | <0.001 |
| 34 | -16 | 20 | Insula Right | 189 | 670.19 | 0.035 | 0.00434 | <0.001 |
| HCs: geographical comparisons overall | | | | | | | | |
| -14 | 24 | 0 | Occipital Cortex | 25526 | 3228.16 | <0.001 | <0.001 | <0.001 |
| 30 | -10 | 46 | Frontal Cortex | 21233 | 1629.81 | <0.001 | <0.001 | <0.001 |
| -28 | -22 | 54 | Precentral Left | 115 | 697.22 | 0.009 | 0.000078 | 0.000026 |
| HCs: geographical comparisons corrected through education | | | | | | | | |
| 32 | -78 | 2 | Occipital Cortex | 22518 | 2476.1 | <0.001 | <0.001 | <0.001 |
| 30 | -10 | 46 | Frontal Cortex | 9102 | 1476.09 | <0.001 | <0.001 | <0.001 |
| 16 | -26 | 6 | Thalamus Left | 6400 | 1474.67 | <0.001 | <0.001 | <0.001 |
| -38 | 14 | 22 | Precentral Left | 964 | 934.57 | 0.003 | <0.001 | <0.001 |
| -44 | 18 | -16 | Orbitofrontal | 169 | 748.36 | 0.009 | <0.001 | <0.001 |
| -44 | 54 | -14 | Orbitofrontal | 155 | 675.87 | 0.017 | <0.001 | <0.001 |
| AD geographical comparisons overall | | | | | | | | |
| 30 | -16 | 36 | Frontal Cortex | 3182 | 1307.17 | <0.001 | <0.001 | <0.001 |
| 10 | 48 | -14 | Paracentral Rigth | 1696 | 1037.58 | 0.001 | <0.001 | <0.001 |
| 30 | -52 | 0 | Hippocampus | 123 | 975.31 | 0.001 | <0.001 | <0.001 |
| -14 | 8 | 34 | Precentral Left | 305 | 897.21 | 0.005 | <0.001 | <0.001 |
| -50 | -12 | -40 | Temporal Pole Left | 1215 | 885.67 | 0.006 | <0.001 | <0.001 |
| 46 | -6 | -32 | Temporal Cortex Rigth | 464 | 879.71 | 0.007 | <0.001 | <0.001 |
| 12 | -58 | 12 | Precuneus | 258 | 738.21 | 0.023 | 0.002403 | <0.001 |
| AD: geographical comparisons corrected through education | | | | | | | | |
| Non results for clusters with more than 100 voxels | | | | | | | | |
| FTLD geographical comparisons overall | | | | | | | | |
| -22 | -24 | -8 | Occipital Cortex | 23169 | 3177.42 | <0.001 | <0.001 | <0.001 |
| 32 | -12 | -44 | Frontal Cortex | 28747 | 2063.84 | <0.001 | <0.001 | <0.001 |
| 36 | -26 | 52 | Postcentral Right | 456 | 821.29 | 0.011 | <0.001 | <0.001 |
| FTLD: geographical comparisons corrected through education | | | | | | | | |
| -22 | -24 | -8 | Occipital Cortex | 21878 | 2592.96 | <0.001 | <0.001 | <0.001 |
| 32 | -8 | -48 | Temporal Cortex | 25048 | 1816.19 | <0.001 | <0.001 | <0.001 |
| 36 | -26 | 52 | Postcentral Right | 428 | 689.35 | 0.016 | <0.001 | <0.001 |

1. Santamaria-Garcia H, Sainz-Ballesteros A, Hernandez H, et al. Factors associated with healthy aging in Latin American populations. *Nature Medicine* 2023; **29**.

2. Moguilner S, García AM, Perl YS, et al. Dynamic brain fluctuations outperform connectivity measures and mirror pathophysiological profiles across dementia subtypes: A multicenter study. *NeuroImage* 2021; **225**: 117522.

3. Esteban O, Birman D, Schaer M, Koyejo OO, Poldrack RA, Gorgolewski KJ. MRIQC: Advancing the automatic prediction of image quality in MRI from unseen sites. *PLoS ONE* 2017; **12**(9).

4. Ganzetti M, Wenderoth N, Mantini D. Intensity inhomogeneity correction of structural MR images: A data-driven approach to define input algorithm parameters. *Frontiers in Neuroinformatics* 2016; **10**: 10.

5. Magnotta VA, Friedman L. Measurement of signal-to-noise and contrast-to-noise in the fBIRN multicenter imaging study. *Journal of Digital Imaging* 2006; **19**(2): 140-7.

6. Power JD, Barnes KA, Snyder AZ, Schlaggar BL, Petersen SE. Spurious but systematic correlations in functional connectivity MRI networks arise from subject motion. *NeuroImage* 2012; **59**(3): 2142-54.
